# Seipin‐Mediated Lipid Droplet Formation in Cardiomyocytes Ameliorates Cardiac Ischemia/Reperfusion Injury

**DOI:** 10.1002/advs.202510203

**Published:** 2025-11-19

**Authors:** Changyun Liu, Junxia Zhang, Yusi Chen, Geng Shen, Yufei Han, Zihao Zhou, Jinxuan Chen, Xuya Kang, Huilin Qu, Jiaxin Duanmu, Haibao Shang, Yingjia Li, Wei Huang, Yan Zhang

**Affiliations:** ^1^ Institute of Cardiovascular Sciences State Key Laboratory of Vascular Homeostasis and Remodeling School of Basic Medical Sciences Peking University Health Science Center Beijing 100191 China; ^2^ Department of Cardiology and Institute of Vascular Medicine Peking University Third Hospital Beijing 100191 China; ^3^ Research Unit of Medical Science Research Management/Basic and Clinical Research of Metabolic Cardiovascular Diseases Haihe Laboratory of Cell Ecosystem Chinese Academy of Medical Sciences Beijing 100191 China; ^4^ Department of Cardiology Beijing Anzhen Hospital Capital Medical University Beijing 100029 China; ^5^ State Key Laboratory of Membrane Biology Institute of Molecular Medicine College of Future Technology Peking University Beijing 100871 China; ^6^ NHC Key Laboratory of Cardiovascular Molecular Biology and Regulatory Peptides Beijing Key Laboratory of Cardiovascular Receptors Research Peking University Beijing 100191 China; ^7^ Institute of Cardiovascular Diseases The First Affiliated Hospital of Dalian Medical University Dalian 116021 China

**Keywords:** cardiomyocyte death, heart, ischemia/reperfusion injury, lipid droplet, seipin

## Abstract

Cardiac ischemia/reperfusion (I/R) injury is an important therapeutic target for ischemic heart disease. Lipid droplets (LDs) are the key organelles involved in lipid metabolism. This study aimed to identify the LD‐mediated protection against lipotoxicity in cardiac I/R injury. LD accumulation is upregulated in hearts subjected to I/R injury; however, it is insufficient to neutralize lipotoxicity or prevent cardiomyocyte death. Seipin played a central role in LD biogenesis in cardiomyocytes following I/R injury. Seipin deficiency led to reduced LD levels and exacerbated cardiac I/R injury. Whereas increased LD levels, via Seipin overexpression or lipolysis inhibition, ameliorated myocardial I/R injury. I/R‐induced downregulation of Seipin is attributed to the reduced expression of its transcription factor USF1, which is required for metabolic adaptation in acute myocardial ischemia. These findings not only elucidate the pathophysiological roles of LDs and Seipin but also provide a promising therapeutic target for myocardial I/R injury.

## Graphical abstract

Lipid droplets (LDs) protect the heart against lipotoxicity in cardiac ischemia/reperfusion (I/R) injury; however, they are insufficient to prevent cardiomyocyte death. Seipin plays a central role in the insufficient formation of LDs, subsequent lipotoxicity, and myocardial injury during cardiac I/R injury. Genetic or pharmacological treatments to improve LDs levels could alleviate I/R‐induced cardiomyocyte death and myocardial injury.

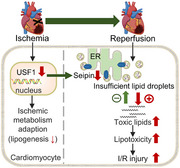

## 1 Introduction

Ischemic heart disease (IHD) is the leading cause of death worldwide.^[^ ^1^, ^2^ ^]^ The main treatment for patients with acute myocardial ischemia is timely and effective restoration of blood flow (i.e., reperfusion) through primary percutaneous coronary intervention,^[^ ^3^ ^]^ coronary artery bypass grafting, or thrombolysis.^[^ ^4^, ^5^ ^]^ However, reperfusion of the ischemic myocardium leads to further irreversible myocardial ischemia/reperfusion (I/R) injury, which causes cardiomyocyte death and permanent tissue damage, consequently worsening the prognosis of IHD.^[^ ^6^, ^7^ ^]^ Currently, an effective clinical means to prevent and treat cardiac I/R injury, which remains an important therapeutic target for IHD, is lacking.

Cardiomyocyte death is an important pathophysiological mechanism underlying cardiac I/R injury.^[^ ^8^ ^]^ Because the proliferative capacity of cardiomyocytes in adult mammals is very limited,^[^ ^9^ ^]^ dead cardiomyocytes cannot be effectively replenished by the division of living cardiac cells, resulting in the permanent loss of cardiac functional units and various serious heart diseases, including depressed cardiac function, arrhythmia and heart failure.^[^ ^10^, ^11^, ^12^ ^]^ The mechanisms underlying cardiac I/R injury include oxidative stress, calcium overload, inflammatory responses, and metabolic disorders.^[^ ^13^ ^]^ However, no effective clinical method that targets myocardial cell death to reduce I/R injury currently exists. Therefore, clarifying the mechanisms of myocardial cell death in I/R injury and reducing cardiomyocyte death are crucial for improving the prognosis and outcome of patients with myocardial ischemia.

Lipids are the main energy source for the adult heart, and lipid oxidation fuels 60%–80% of the cardiac energy demand.^[^ ^14^, ^15^ ^]^ Abnormalities in lipid metabolism play an important role in various heart diseases, including diabetic heart disease, cardiac fibrosis, and heart failure.^[^ ^16^, ^17^ ^]^ The dysregulation of energy metabolism in the heart is pivotal in the development of I/R injury.^[^ ^18^, ^19^ ^]^ In the ischemic myocardium, anaerobic oxidation is the main energy source for cells owing to a lack of oxygen. During reperfusion, fatty acid oxidation recovers rapidly and suppresses aerobic respiration and Oxidative phosphorylation (OXPHOS).^[^ ^20^ ^]^ The influx of oxygen into hypoxic tissues leads to rapid activation of the electron transport chain and production of high levels of reactive oxygen species (ROS).^[^ ^21^ ^]^ Intracellular levels of free fatty acids (FFA) increase because of elevated blood lipids and the breakdown of phospholipid membranes. FFA oversupply may overwhelm the mitochondrial capacity to oxidize FFAs for adenosine triphosphate (ATP) production, leading to the accumulation of toxic lipid intermediates such as diacylglycerol (DAG) and ceramide, which directly induce lipotoxicity in cardiomyocytes.^[^ ^15^ ^]^ Lipotoxicity is involved in the pathophysiology of various cardiovascular diseases, including heart failure,^[^ ^22^ ^]^ diabetic cardiomyopathy,^[^ ^23^, ^24^ ^]^ and pulmonary arterial hypertension.^[^ ^25^, ^26^ ^]^ Enhancing cardiac triacylglycerol (TAG) metabolism to reduce lipotoxicity can protect the heart against ischemic stress.^[^ ^27^ ^]^ However, the mechanisms underlying lipotoxicity in cardiac I/R injury remain unclear. Similarly, there is a lack of effective clinical approaches to prevent and treat cardiac I/R injury by targeting lipotoxicity.

Lipid droplets (LDs) are dynamic organelles with a cycle of biogenesis and consumption responsible for lipid storage and mobilization.^[^ ^28^, ^29^ ^]^ LDs are synthesized in the endoplasmic reticulum (ER) and composed of neutral lipid cores, mainly TAGs and sterol esters, surrounded by phospholipid monolayers.^[^ ^30^ ^]^ When lipid uptake exceeds consumption, excess FFA are transported to the ER and used to synthesize neutral lipids under the action of various enzymes. Following the accumulation of neutral lipids, the LDs bud from the ER, grow until maturity, and detach from the ER.^[^ ^31^ ^]^ LD‐associated proteins (LDAPs) are proteins either on the surface of LDs or in the cytosol that contribute to LDs and lipid metabolism, including Seipin and FIT related to LDs biogenesis, PLINs on the surface of LDs, and glycerol‐3‐phosphate acyltransferases (GPATs) related to lipid synthesis.^[^ ^29^ ^]^ The storage and flow of LDs in the heart are closely related to heart function.^[^ ^32^ ^]^ LD levels increase in cardiomyocytes during I/R,^[^ ^33^, ^34^ ^]^ although their functions as well as the underlying mechanisms of LDs in I/R injury remain unclear.

Here, we found that myocardial LDs were increased by I/R injury to neutralize lipotoxicity but were insufficient to prevent cardiomyocyte death and eliminate cardiac damage induced by I/R injury. Seipin, a key factor for LDs and lipid homeostasis, played an essential role in LD formation and was downregulated in cardiomyocytes in acute I/R injury. Seipin overexpression further increased LD levels in cardiomyocytes and alleviated cardiac I/R injury. The decrease in Seipin in acute ischemia was attributable to the downregulation of upstream stimulatory factor 1 (USF1), which is a metabolic adaptation in response to myocardial ischemia. We not only identified the role of LDs in cardiac I/R injury and their underlying mechanisms, but also identified a new therapeutic target for IHDs.

## 2 Results

### 2.1 LD Upregulation in the Hearts with I/R Injury

**Figure 1:**
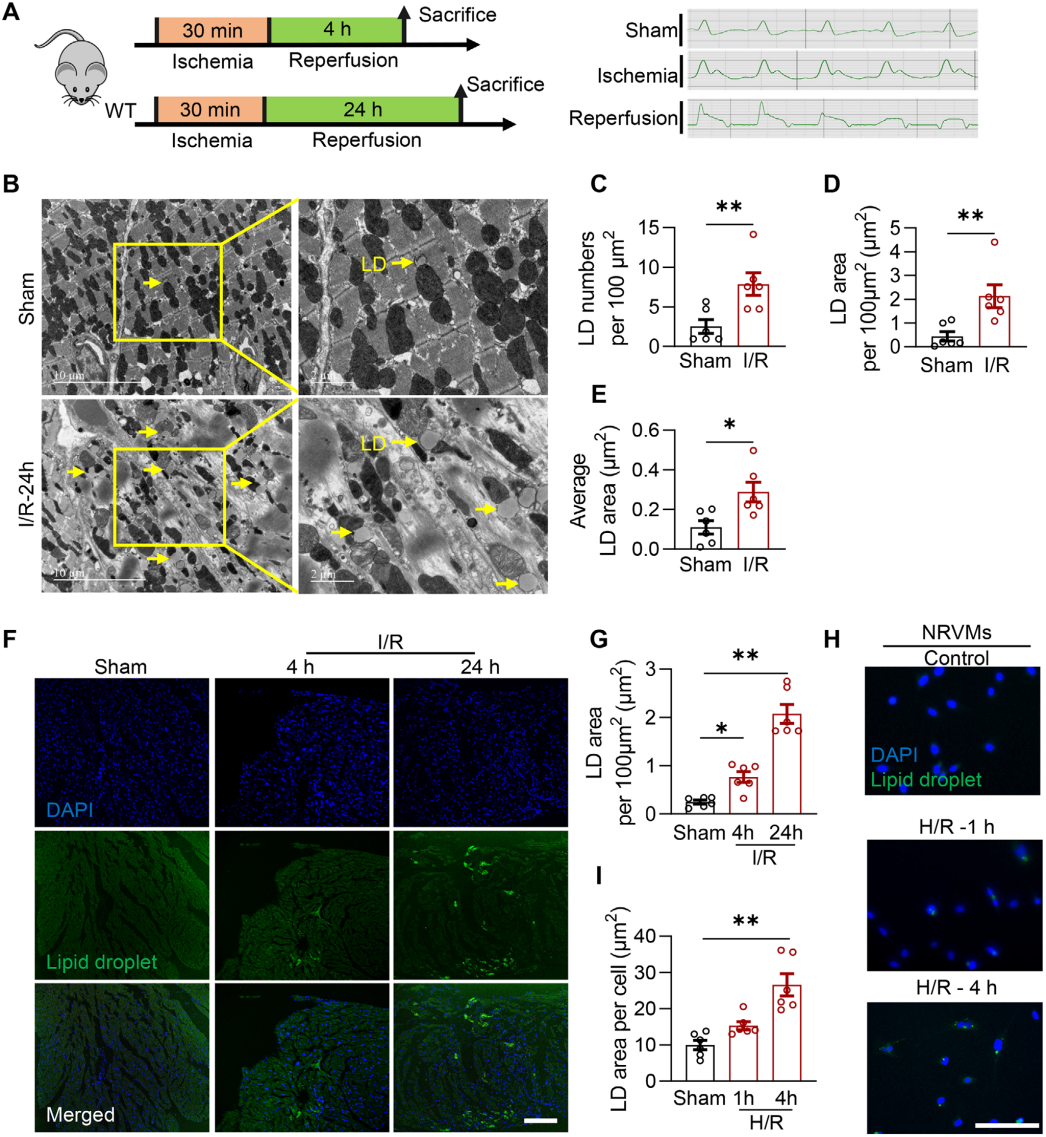
Excessive LDs were accumulated in the hearts with I/R injury. A) Diagram of the experimental protocols; B) Representative TEM images of the myocardium in WT mice with or without I/R injury (30‐min ischemia followed by 24‐h reperfusion), LDs were labeled with yellow arrows; C‐E) Quantitative analysis of LD numbers per 100 µm^2^ (C), total LD area per 100 µm^2^ (D) and average LD area (E) based on TEM images in panel B (*n* = 6 each group); F) Representative fluorescence images indicating LDs labeled with Bodipy 493/503 (green) of myocardium in WT mice with or without I/R injury (30‐min ischemia followed by 4 and 24‐h reperfusion; scale bar, 100 µm); G) Quantitative analysis of positive area of Bodipy 493/503 staining per 100 µm^2^ in panel F (*n* = 6 each group); H) Representative Bodipy 493/503 images of cultured NRVMs with or without H/R insults (6‐h hypoxia followed by 1 and 4‐h reoxygenation; scale bar, 100 µm); I) Quantitative analysis of positive area of Bodipy 493/503 staining per cell in panel H (*n* = 6 each group). Data are expressed as Mean ± SEM. Statistical analysis was performed by Student's *t*‐test (C, D and E) and one‐way *ANOVA* followed by Tukey's post‐hoc tests (G and I). *P < 0.05, **P < 0.01.

First, we examined the changes in LDs in the ischemic area of mouse hearts after I/R injury (30 min of ischemia followed by 4 or 24 h of reperfusion, **Figure** **1**A). The electrocardiogram (ECG) results demonstrated the successful construction of the mice cardiac I/R model. Cardiac LDs were detected using transmission electron microscopy (TEM) and Bodipy 493/503 staining as previously described.^[^ ^35^ ^]^ TEM revealed a marked increase in cardiac LDs in the hearts with I/R damage (Figure 1B). Specifically, the LD number, total LD area, and average LD area increased dramatically in the hearts of I/R mice compared with those in sham animals (Figure 1C–E). The I/R‐induced increase in myocardial LDs content was confirmed using Bodipy 493/503 staining (Figure 1F,G).

To further test the LD accumulation in cardiomyocytes, hypoxia/reoxygenation (H/R) in neonatal rat ventricular cardiomyocytes (NRVMs) was used to mimic in vivo cardiac I/R injury.^[^ ^36^, ^37^ ^]^ Bodipy 493/503 staining demonstrated that LDs were markedly upregulated upon H/R (6 h of hypoxia followed by 1 or 4 h of reoxygenation, Figure 1H,I) in NRVMs. Overall, I/R caused cardiac injury accompanied by myocardial LD upregulation.

### 2.2 Seipin was Essential for LD Formation in the Hearts Induced by I/R Injury

**Figure 2:**
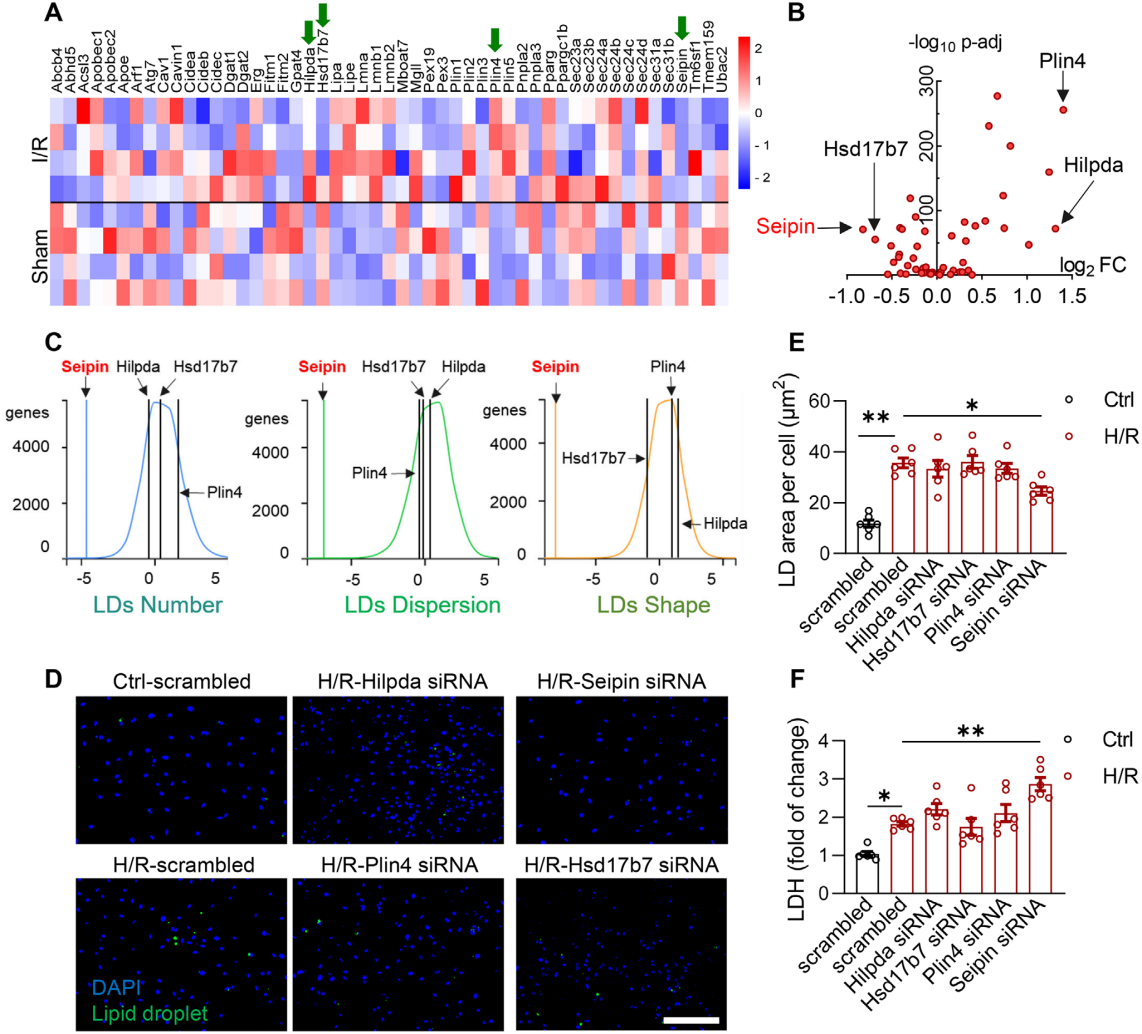
Seipin is essential for LDs formation in the hearts with I/R injury. A,B) Heatmap (A) and volcano plot (B) of the changes of the mRNA levels of LD‐associated proteins from the ischemic myocardial tissue in WT mice with or without I/R injury (30‐min ischemia followed by 4‐h reperfusion, n = 4 each group); C) The phenotypes of the knockdown of LD‐associated proteins on LD parameters from *Lipid Droplet Knowledge Portal* (https://lipiddroplet.org) database; D,E) Representative fluorescence images (D) indicating LDs labeled with Bodipy 493/503 of NRVMs with or without H/R insults (6‐h hypoxia followed by 4‐h reoxygenation; scale bar, 200 µm), together with the quantitative analysis (E; n = 6 each group); F) LDH concentrations in culture medium of the NRVMs transfected with scrambled or siRNA of Hilpda, Hsd17b7, Plin4 and Seipin with or without H/R insults (6‐h hypoxia followed by 4‐h reoxygenation; n = 6 each group). Data are expressed as Mean ± SEM. Statistical analysis was performed by one‐way *ANOVA* (E and F) followed by Tukey's post‐hoc tests. *P < 0.05, **P < 0.01.

Next, we investigated the mechanisms underlying the changes in LDs and their potential regulatory targets during myocardial I/R. Based on RNA‐seq transcriptome data from the ischemic heart tissues of mice with acute myocardial I/R injury (30 min of ischemia followed by 4 h of reperfusion),^[^ ^36^ ^]^ we investigated the changes in LDAPs that are essential for maintaining the normal function of LDs.^[^ ^29^, ^38^ ^]^ Among them, the expressions of Seipin, Hsd17b7, Plin4, and Hilpda were the most significantly altered (**Figure** **2**A,B).

To identify the proteins responsible for LD formation during cardiac I/R injury, we examined the effects of these four genes on LDs using the *Lipid Droplet Knowledge Portal (lipiddroplet.org)* database.^[^ ^39^, ^40^, ^41^ ^]^ This portal includes a genome‐wide screening of the results of RNAi screening, RNA‐seq, and proteomics in THP‐1 and SUM159 cells to identify genes affecting LDs.^[^ ^40^ ^]^ Knocking down Seipin resulted in a reduction in the number, abnormal distribution, and shape of intracellular LDs in THP‐1 macrophages (Figure 2C). Thus, Seipin may play an essential role in the growth and maintenance of LDs. Furthermore, to investigate the role of these four genes in cardiomyocytes, we compared their effects on LD formation and cell death in NRVMs induced by H/R. Seipin silencing significantly reduced LDs levels in cardiomyocytes subjected to H/R (Figure 2D,E; Figure S1, Supporting Information). H/R‐induced cardiomyocyte death (indexed by the lactate dehydrogenase [LDH] concentration in the medium) was also exaggerated by Seipin silencing (Figure 2F). Thus, Seipin may play an essential role in LD formation and cell survival in cardiomyocytes during I/R injury.

### 2.3 Seipin Deficiency Aggravated Cardiac I/R Injury

**Figure 3:**
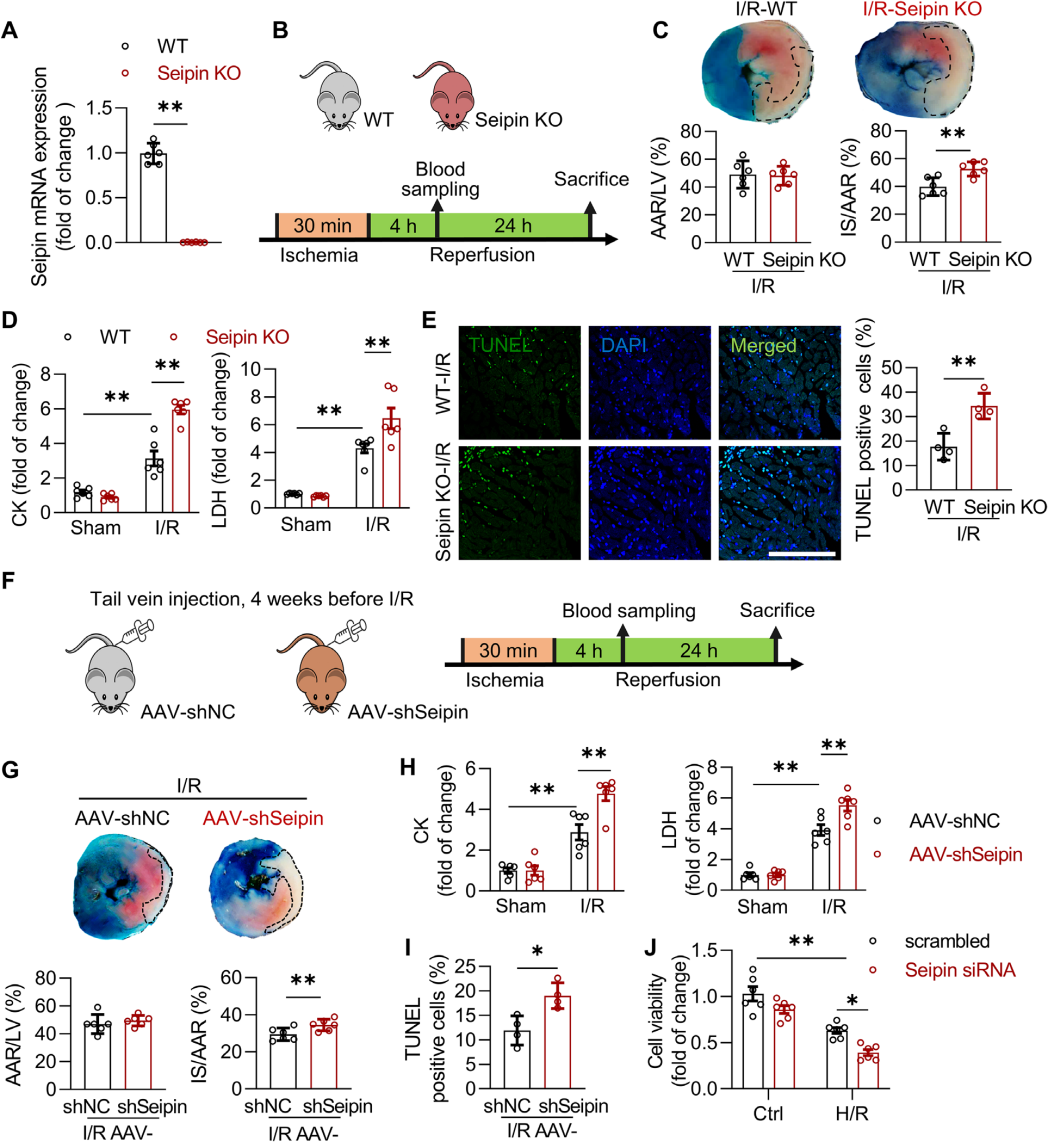
Deficiency of Seipin aggravates cardiac I/R injury. A) The mRNA level of Seipin in the hearts of 10‐week‐old WT and Seipin KO mice detected by qPCR (*n* = 6 each group); B) Diagram of the experimental protocols for C–E; C) Representative cardiac tissue sections stained with Evans blue/TTC and quantification of cardiac infarct size of WT and Seipin KO mice subjected to I/R injury (30‐min ischemia followed by 24‐h reperfusion). The non‐ischemic area was stained in blue by Evans blue, the ischemic area at risk (AAR) was stained in red by TTC and the infarcted area (IS) appeared white (surrounded by black lines, n = 6 each group); D) Plasma CK and LDH levels of WT and Seipin KO mice with or without to I/R injury (30‐min ischemia followed by 4h reperfusion, n = 6 each group); E) Representative myocardial sections subjected to the TUNEL‐staining (scale bar, 100 µm) and quantification of the ischemic area of WT and Seipin KO mice with I/R injury (30‐min ischemia followed by 24‐h reperfusion, n = 4 each group). TUNEL‐positive cells are shown in green. The DAPI‐stained nuclei are shown in blue; F) Diagram of the experimental protocols for G‐I; G) Representative cardiac tissue sections stained with Evans blue/TTC and quantification of cardiac infarct size of AAV‐shNC and AAV‐shSeipin mice subjected to I/R injury (30‐min ischemia followed by 24‐h reperfusion; n = 6 each group); H) Plasma CK and LDH levels of AAV‐shNC and AAV‐shSeipin mice with or without to I/R injury (30‐min ischemia followed by 4‐h reperfusion, n = 6 each group); I) Quantification TUNEL‐staining of the ischemic area of AAV‐shNC and AAV‐shSeipin mice with I/R injury (30‐min ischemia followed by 24‐h reperfusion, n = 4 each group); J) The cell viability assayed by cellular CCK‐8 in NRVMs transfected with scrambled or Seipin siRNA for 48 h with or without H/R insults (6‐h hypoxia followed by 4‐h reoxygenation; n = 6 each group). Data are expressed as Mean ± SEM. Statistical analysis was performed by Student's *t*‐test (C, E, G and I), Mann‐Whitney *U* test (A), and two‐way *ANOVA* (D, H and J) followed by Tukey's post‐hoc tests. *P < 0.05, **P < 0.01.

Seipin plays important roles in adipocyte differentiation and lipid homeostasis.^[^ ^42^ ^]^ Its absence results in generalized lipodystrophy, which is closely associated with numerous metabolic syndromes.^[^ ^43^ ^]^ Seipin is a key protein in the assembly of LDs and their maintenance at ER–LD junctions for the exchange of proteins and lipids.^[^ ^44^ ^]^ Seipin‐knockout (KO) mice, constructed by homologous recombination as described (**Figure** **3**A),^[^ ^45^, ^46^ ^]^ were used to investigate the role of Seipin in myocardial I/R injury in vivo. Global Seipin KO mice develop heart failure with preserved ejection fraction in the aged stages, while exhibiting no cardiac pathology in the youth.^[^ ^47^ ^]^ In this study, 10‐week‐old mice were used for baseline assessments and cardiac function evaluations. Based on echocardiography, no difference in cardiac morphology or function was observed between Seipin KO mice and their wild type (WT) littermates at 10 weeks of age (Figure S2A,B, Supporting Information). Body weight and blood lipid levels were not affected by Seipin KO in mice (Figure S2C, Supporting Information), suggesting that Seipin deficiency did not elicit evident changes in hearts under normal conditions at 10 weeks of age.

Next, the role of Seipin was investigated in WT and Seipin KO mice with myocardial I/R injury (30 min of ischemia followed by 24 h of reperfusion, Figure 3B). Cardiac Evans blue/triphenyltetrazolium chloride (TTC) staining was performed at the end of reperfusion to show the non‐ischemic area (stained blue by Evans blue), the ischemic area at risk (AAR, stained red by TTC), and the infarcted area (IS, white). Although the ischemic size indexed by AAR/LV was similar between these two groups, the infarction size (IS/AAR) was markedly increased in Seipin KO mice compared to that in WT mice (Figure 3C). Similarly, cardiomyocyte necrosis (indexed by plasma LDH and creatine kinase [CK] levels, Figure 3D) and apoptosis (indexed by TUNEL staining, Figure 3E) were exaggerated in Seipin KO mice compared to that in WT mice.

We also used a cardiac‐specific Seipin‐knockdown model by injecting adeno‐associated virus 9 (AAV9)‐cardiac troponin T (cTNT)‐shSeipin (Figure 3F; Figure S2D–F, Supporting Information). Consistent with our findings in global Seipin KO mice, the myocardial infarction size (Figure 3G), cardiomyocyte necrosis (indexed by plasma LDH and CK levels, Figure 3H), and apoptosis (indexed by TUNEL staining, Figure 3I) were markedly increased by the cardiac‐specific inhibition of Seipin. This result was confirmed in cultured NRVMs with H/R injury. With siRNA targeting Seipin, which downregulated cellular Seipin expression (Figure S1A, Supporting Information), reduced cell viability (detected by Cell Counting Kit 8) induced by H/R was more severe than that in the scrambled group (Figure 3J). Thus, Seipin deficiency aggravated cardiac I/R injury.

### 2.4 Seipin Overexpression Alleviated Cardiac I/R Injury

**Figure 4:**
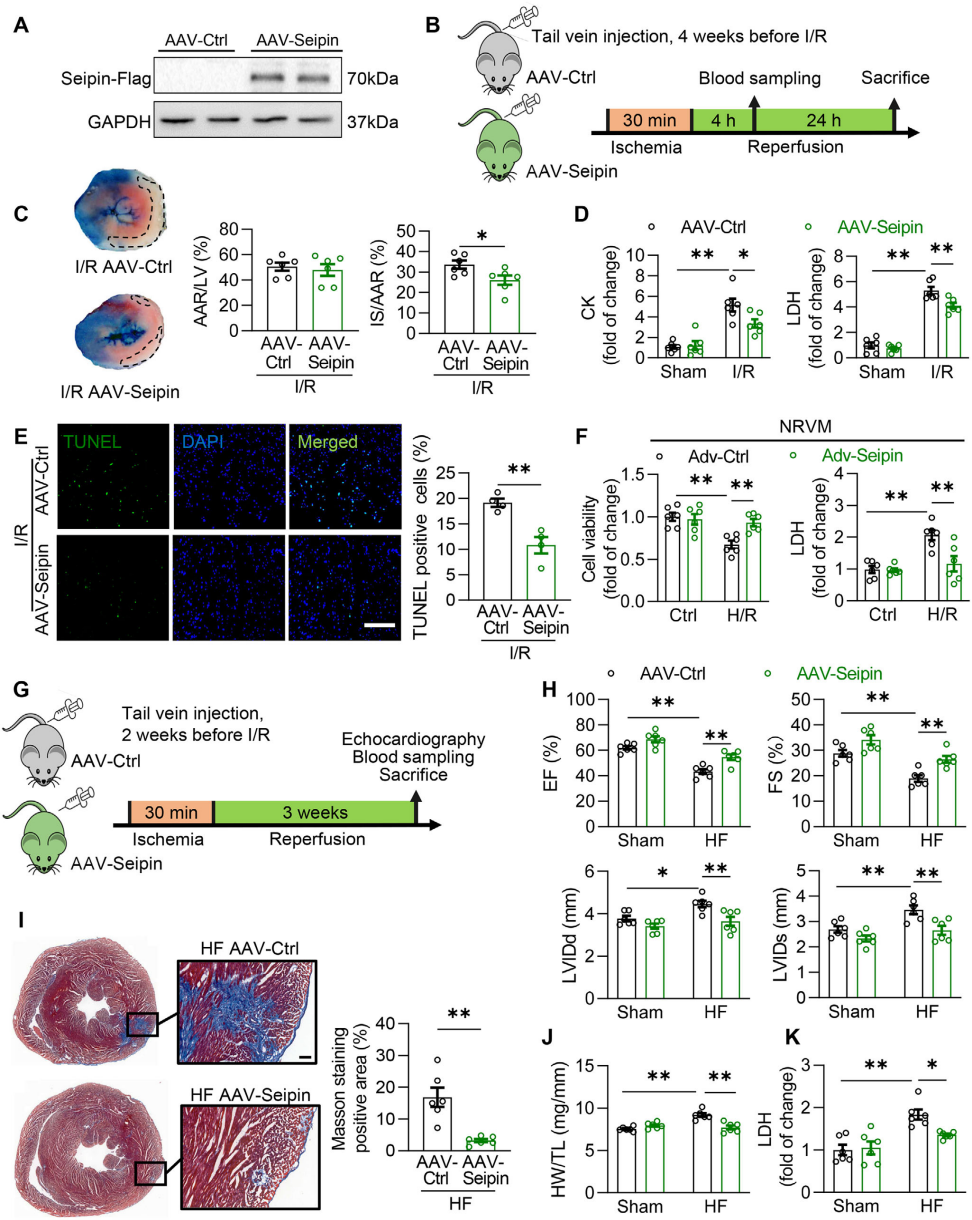
Overexpression of Seipin alleviates cardiac I/R injury. A) Representative western blotting images of Seipin‐Flag protein levels of the hearts from AAV‐Ctrl or AAV‐Seipin mice; B) Diagram of the experimental protocols for C–E; C) Representative cardiac tissue sections stained with Evans blue/TTC and quantification of cardiac infarct size of AAV‐Ctrl and AAV‐Seipin mice subjected to I/R injury (30‐min ischemia followed by 24‐h reperfusion, n = 6). The non‐ischemic area was stained in blue by Evans blue, the ischemic area at risk (AAR) was stained in red by TTC and the infarcted area (IS) appeared white (surrounded by black lines); D) Plasma CK and LDH levels of AAV‐Ctrl and AAV‐Seipin mice with or without to I/R injury (30‐min ischemia followed by 4‐h reperfusion, n = 6 each group); E) Representative myocardial sections subjected to the TUNEL‐staining (scale bar, 100 µm) and quantification of the the ischemic area of AAV‐Ctrl and AAV‐Seipin mice with I/R injury (30‐min ischemia followed by 24‐h reperfusion, n = 4 each group). TUNEL‐positive cells are shown in green. The DAPI‐stained nuclei are shown in blue; F) The cell viability assayed by cellular CCK‐8 and LDH concentration in culture medium in NRVMs infected with Adv‐Ctrl or Adv‐Seipin at 10 MOI for 48 h with or without H/R insults (6‐h hypoxia followed by 4‐h reoxygenation; n = 6 each group); G) Diagram of the experimental protocols for H–K; H‐K) Left ventricular morphology and function evaluated by echocardiography (H), cardiac gross morphology by masson staining (I; scale bar, 100 µm), the ratio of heart weight to tibia length (J, HW/TL ratio), plasma LDH levels (K) of AAV‐Ctrl and AAV‐Seipin mice subjected to I/R injury (30‐min ischemia followed by 3‐week reperfusion, n = 6 each group). MOI, multiplicity of infection. Data are expressed as Mean ± SEM. Statistical analysis was performed by Student's *t*‐test (C and E), Mann–Whitney *U* test (I) and two‐way *ANOVA* (D, F, H, J and K) followed by Tukey's post‐hoc tests. *P < 0.05, **P < 0.01.

To further determine the role of Seipin in cardiac I/R injury, we overexpressed Seipin in mouse cardiomyocytes via tail vein injection of AAV9, in which full‐length human Seipin was expressed through the cTNT promoter (defined as AAV‐Seipin). Mice injected with AAV9‐Ctrl served as controls (AAV‐Ctrl). Seipin overexpression in the heart was confirmed by western blotting (**Figure** **4**A). Body weight, cardiac function, and blood lipids were not affected by Seipin overexpression in the heart of mice (Figure S3A–D, Supporting Information), confirming the safety of Seipin upregulation in the hearts.

In response to I/R injury, the myocardial infarction size (Figure 4B,C), cardiomyocyte necrosis (indexed by plasma LDH and CK levels, Figure 4D), and apoptosis (indexed by TUNEL staining, Figure 4E) were markedly alleviated by Seipin overexpression in cardiomyocytes. Furthermore, the protective effects of Seipin against myocardial I/R injury were confirmed in cultured cardiomyocytes. In cultured NRVMs, adenovirus‐mediated Seipin overexpression (Figure S3E, Supporting Information) markedly reduced H/R‐induced cardiomyocyte death (Figure 4F), suggesting that Seipin overexpression alleviated cardiac I/R (H/R) injury.

Subsequently, we evaluated the role of Seipin in I/R‐induced heart failure. In the mouse heart failure model induced by 30‐min cardiac ischemia followed by 3‐week reperfusion (Figure 4G), Seipin overexpression in cardiomyocytes alleviated cardiac dysfunction and remodeling (Figure 4H–J). Furthermore, Seipin overexpression decreased cardiomyocyte death in the heart following chronic I/R injury (Figure 4K). Therefore, upregulation of Seipin alleviated not only acute myocardial I/R injury but also subsequent heart failure.

### 2.5 Seipin Promoted LD Formation and Improved Lipid Metabolism in the Hearts With I/R Injury

**Figure 5:**
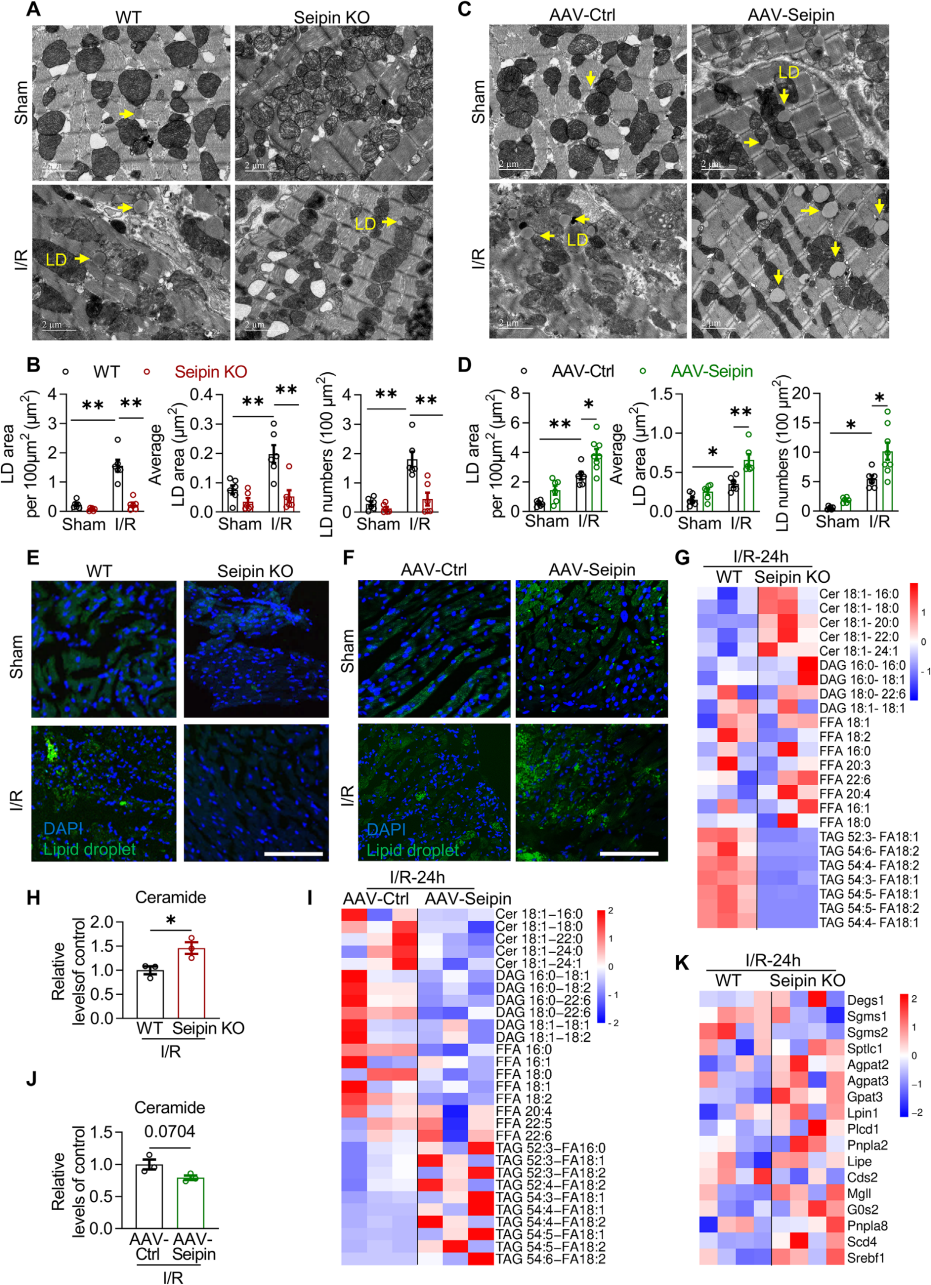
Seipin promotes LDs formation and improve lipid metabolism in the hearts with I/R injury. A) Representative TEM images in myocardium of WT and Seipin KO mice with or without I/R injury (30‐min ischemia followed by 24‐h reperfusion). LDs were labeled with yellow arrows; B) Quantitative analysis of total LD area per 100 µm^2^, average LD area and LD numbers per 100 µm^2^ based on TEM images in panel A (*n* = 6 each group); C) Representative TEM images in the myocardium of AAV‐Ctrl and AAV‐Seipin mice with or without I/R injury (30‐min ischemia followed by 24‐h reperfusion), LDs were labeled with yellow arrows; D) Quantitative analysis of total LD area per 100 µm^2^, average LD area and LD numbers per 100 µm^2^ based on TEM images in panel C (*n* = 6 each group); E,F) Representative fluorescence images indicating LDs labeled with Bodipy 493/503 and nucleus labeled with DAPI in myocardium of WT/Seipin KO mice (E) and AAV‐Ctrl/AAV‐Seipin mice (F) with or without I/R injury (30‐min ischemia followed by 24‐h reperfusion; scale bar, 100 µm); G,H) Heatmap of metabolic alterations organized by lipid class (G) and quantitative analysis of ceramide (H) in myocardium of WT and Seipin KO mice subjected to I/R injury (30‐min ischemia followed by 24‐h reperfusion; n  =  3 each group). I,J) Heatmap of metabolic alterations organized by lipid class (I) and quantitative analysis of ceramide (J) in myocardium of AAV‐Ctrl and AAV‐Seipin mice subjected to I/R injury (30‐min ischemia followed by 24‐h reperfusion, n = 3 each group); K) Heatmap of lipid metabolism‐related enzymes (*n* = 4 each group) of RNA‐seq data in the hearts of WT and Seipin KO mice with or without I/R injury. Data are expressed as Mean ± SEM. Statistical analysis was performed by Student's *t*‐test (H and J) and two‐way *ANOVA* (B and D) followed by Tukey's post‐hoc tests. *P < 0.05, **P < 0.01.

To explore the pathophysiological mechanisms of Seipin‐mediated cardio‐protection against I/R injury, TEM and Bodipy 493/503 staining were used to detect LD levels. Cardiac LD formation was markedly impaired in response to I/R in Seipin KO mice (**Figure** **5**A). Specifically, TEM data demonstrated that the total LD area, average LD area, and LD number were decreased in Seipin KO mice after myocardial I/R injury (Figure 5B). By contrast, Seipin overexpression promoted LD formation during I/R injury (Figure 5C,D). The TEM results were also confirmed by Bodipy 493/503 staining, including the results from Seipin KO mice (Figure 5E; Figure S4A, Supporting Information) and the mice with Seipin overexpression (Figure 5F; Figure S4B, Supporting Information). Furthermore, Seipin knockdown reduced the LD levels in cultured NRVMs subjected to H/R injury (Figure S4C, Supporting Information). Seipin overexpression promoted LD formation in cultured cardiomyocytes (Figure S4D, Supporting Information) subjected to H/R damage. Thus, Seipin could be both sufficient and necessary for myocardial LD biogenesis during I/R injury.

LDs reduce cell lipotoxicity through transient storage of excess FFAs, alleviation of cellular stress, and maintenance of energy homeostasis.^[^ ^30^ ^]^ Next, we investigated the effects of Seipin on myocardial lipid metabolism in response to I/R injury. Based on the lipidomic analysis of heart tissue, Seipin KO increased toxic lipids in the heart after I/R, including ceramide and FFA (Figure 5G,H; Figure S4E,F, Supporting Information). TAG levels were also significantly decreased (Figure S4F, Supporting Information), indicating the role of Seipin in maintaining neutral lipid pools. By contrast, AAV‐mediated Seipin overexpression markedly decreased toxic lipid levels in mouse hearts subjected to I/R injury (Figure 5I,J; Figure S4G,H, Supporting Information). In addition, transcriptome sequencing results revealed that Seipin deficiency aggravated disorders of lipid metabolism‐related pathways and enzymes in hearts subjected to I/R injury. Kyoto Encyclopedia of Genes and Genomes (KEGG) pathway enrichment analysis revealed significant clustering of differentially expressed genes within lipid metabolic pathways, particularly those governing sphingolipid metabolism, glycerolipid turnover, and fatty acid biosynthesis (Figure S4I, Supporting Information). The decreased expression of Sgms1/2 was consistent with ceramide accumulation, whereas Agpat2/3 and Gpat3 upregulation may explain the increase in DAG levels. Moreover, Srebf1 and Scd4, key transcriptional and enzymatic regulators of fatty acid desaturation, were markedly increased, suggesting an increase in fatty acid unsaturation (Figure 5K), which aligns with the results of the KEGG pathway analysis. Overall, Seipin was critical for LD formation and improved lipid metabolism in cardiomyocytes during myocardial I/R injury.

### 2.6 Seipin was Essential for the Protection of LDs Against I/R‐Induced Cardiac Lipotoxicity and the Consequent Pathological Response

**Figure 6:**
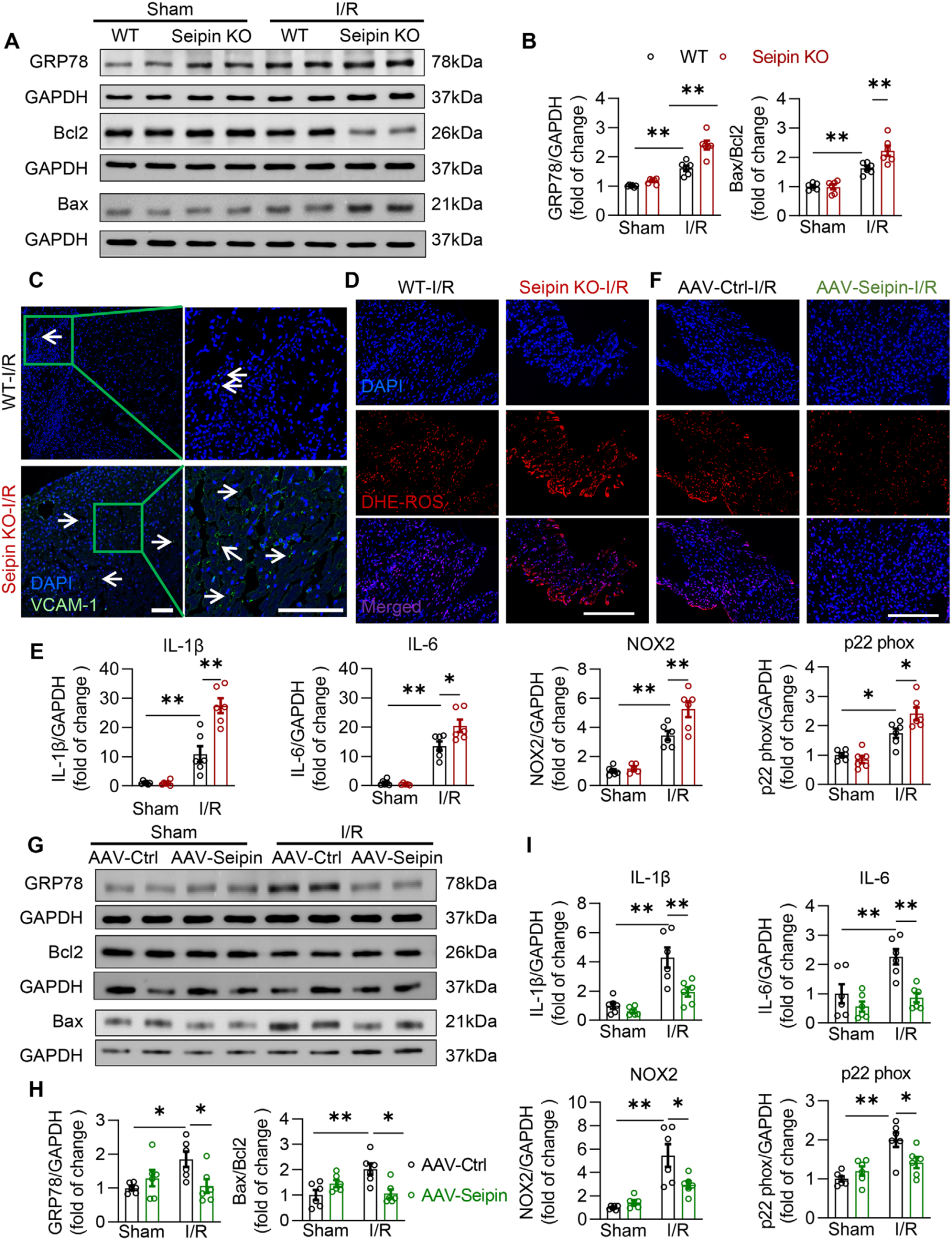
Seipin is essential for I/R‐induced lipotoxicity and its related cardiac pathology. A‐B) Representative western blots images (A) and quantitative analysis (B, n = 6 each group) of GRP78, Bcl2 and Bax in myocardium of WT and Seipin KO mice with or without I/R injury (30‐min ischemia followed by 24‐h reperfusion); C) Representative myocardial sections subjected to the VCAM1‐staining in myocardium of WT and Seipin KO mice with I/R injury (30‐min ischemia followed by 24‐h reperfusion). VCAM1‐positive cells are shown in green. The DAPI‐stained nuclei are shown in blue (scale bar, 50 µm); D) The representative myocardial sections subjected to DHE staining in WT and Seipin KO mice subjected to I/R injury (30‐min ischemia followed by 24‐h reperfusion). The DHE‐stained ROS are shown in red. The DAPI‐stained nuclei are shown in blue (scale bar, 100 µm); E) The mRNA levels of IL‐1β, IL‐6, NOX2 and p22 phox detected by qPCR in myocardium of WT and Seipin KO mice with or without I/R injury (30‐min ischemia followed by 24‐h reperfusion; n = 6 each group); F) The representative myocardial sections subjected to DHE staining in AAV‐Ctrl and AAV‐Seipin mice subjected to I/R injury (30‐min ischemia followed by 24‐h reperfusion). The DHE‐stained ROS are shown in red. The DAPI‐stained nuclei are shown in blue (scale bar, 100 µm); G–I) Representative western blotting images (G) and quantitative analysis (H) of GRP78, Bcl2 and Bax (*n* = 6 each group), and mRNA levels of IL‐1β, IL‐6, NOX2 and p22 phox (I) in myocardium of AAV‐Ctrl and AAV‐Seipin mice with or without I/R injury (30‐min ischemia followed by 24‐h reperfusion; n = 6 each group). Data are expressed as Mean ± SEM. Statistical analysis was performed by two‐way *ANOVA* (B, E, H and I) followed by Tukey's post‐hoc tests. *P < 0.05, **P < 0.01.

Lipotoxicity is a pathological process in which excessive accumulation of cytotoxic lipids (such as FFA, DAG, and ceramides) within cells leads to cell dysfunction and death through the induction of inflammatory activation, oxidative stress, and ER stress.^[^ ^48^, ^49^ ^]^ Therefore, we investigated the role of Seipin in the lipotoxicity‐related cardiac pathology. Seipin deficiency aggravated I/R‐induced ER stress, apoptosis, ROS production, and inflammation in cardiomyocytes. Specifically, compared with WT mice, the protein levels of the ER stress marker GRP78 were further increased in Seipin KO mice after I/R, accompanied by an increase in apoptosis‐related proteins, the ratio of Bcl2‐associated X (Bax) to B‐cell lymphoma‐2 (Bcl2) (**Figure** **6**A,B). In addition, the inflammation (indexed by VCAM‐1 staining and the levels of inflammatory factors IL‐1β and IL‐6) and ROS levels (indexed by DHE staining, the levels of pro‐oxidative factors nicotinamide adenine dinucleotide phosphate (NADPH oxidase 2 (NOX2) and p22 phox) were exaggerated in Seipin KO mice with I/R injury (Figure 6C–E). These results were further confirmed in cultured NRVMs with H/R injury, indicating that Seipin knockdown aggravated lipotoxicity‐related cell damage (Figure S5A,B, Supporting Information). By contrast, Seipin overexpression protected the heart against I/R‐induced excessive ROS production, ER stress, and inflammation, as well as the consequent cardiomyocyte death (Figure 6F–I), which was phenocopied in NRVMs with Seipin overexpression subjected to H/R injury (Figure S5C,D, Supporting Information). Therefore, Seipin was essential for the protection of LDs against I/R‐elicited myocardial lipotoxicity and the consequent cardiac pathology.

### 2.7 Upregulation of LD Levels Alleviated Cardiac I/R Injury

**Figure 7:**
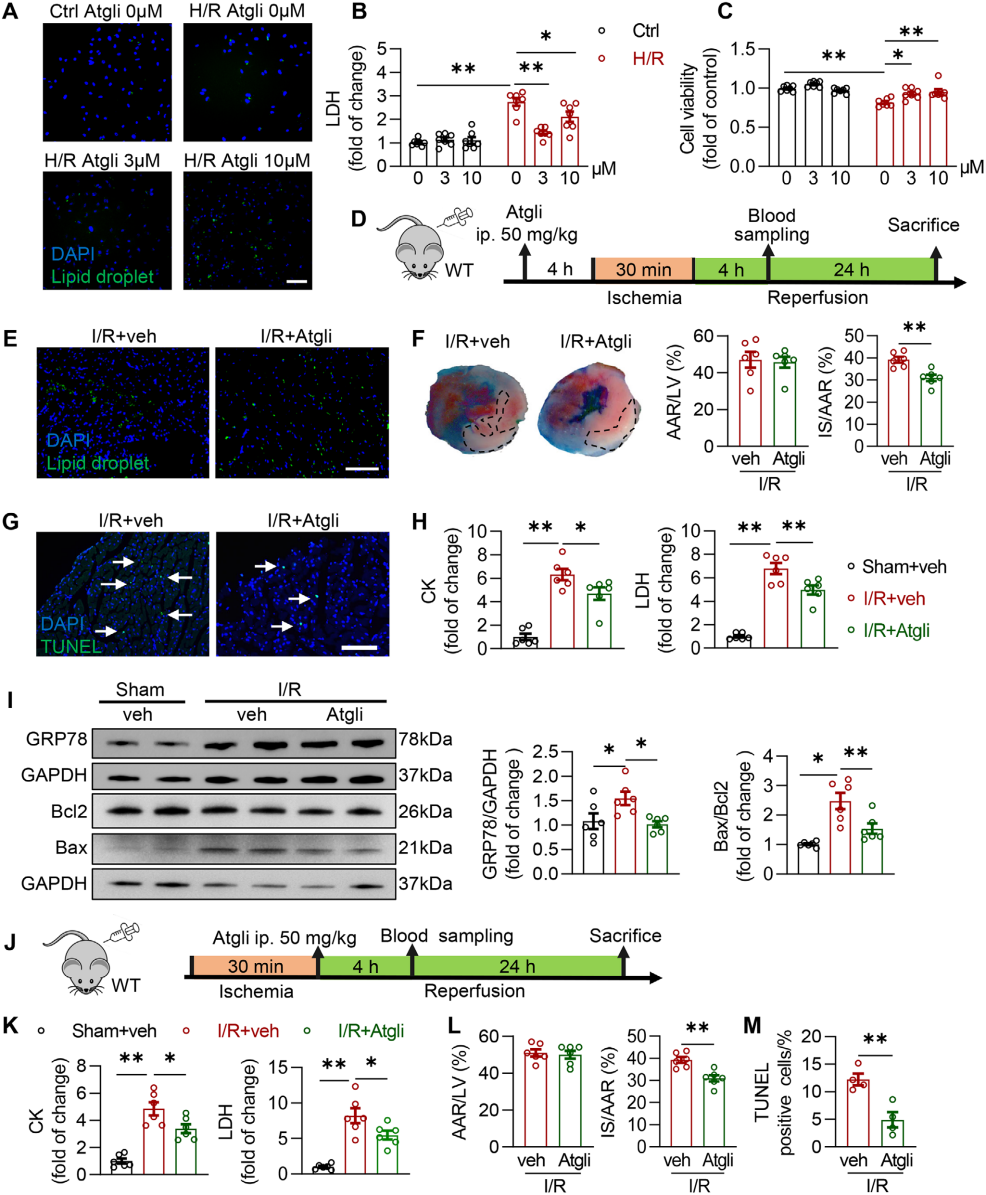
Upregulation of LDs levels alleviates cardiac I/R injury. A–C) Representative fluorescence images indicating LDs labeled with Bodipy 493/503 and nucleus labeled with DAPI (A, scale bar, 100 µm), LDH concentration in culture medium (B) and cell viability assayed by CCK‐8 (C) in NRVMs treated with vehicle or Atglistatin with or without H/R insults (6‐h hypoxia followed by 4‐h reoxygenation; n = 7 each group). Atglistatin was add to the medium 1 h before hypoxia; D) Diagram of the experimental protocols for E‐I, Atglistatin or an equal volume of vehicle solution was given to mice (50 mg kg^−1^) by *i.p*. injection 4 h before I/R surgery or sham operation; E) Representative fluorescence images indicating LDs labeled with Bodipy 493/503 and nucleus labeled with DAPI in the myocardium WT mice subjected to I/R injury (30‐min ischemia followed by 24‐h reperfusion) with vehicle or Atglistatin treatment (50 mg kg^−1^, *i.p*., 4 h before I/R surgery; scale bar, 100 µm); F) Representative cardiac tissue sections stained with Evans blue/TTC and quantification of cardiac infarct size in the heart of WT mice subjected to I/R injury (30‐min ischemia followed by 24‐h reperfusion) with vehicle or Atglistatin treatment (50 mg kg^−1^, *i.p*., 4 h before I/R surgery). The non‐ischemic area was stained in blue by Evans blue, the ischemic area at risk (AAR) was stained in red by TTC and the infarcted area (IS) appeared white (surrounded by black dashed lines; n = 6 each group); G–I) Representative myocardial sections subjected to TUNEL staining labeled with white arrows (G; scale bar, 100 µm), plasma CK and LDH levels (H, n = 6 each group), representative western blotting images and quantitative analysis of GRP78, Bcl2 and Bax (I, n = 6 each group) in the hearts of WT mice with or without I/R injury (30‐min ischemia followed by 24‐h reperfusion) with vehicle or Atglistatin treatment (50 mg kg^−1^, *i.p*., 4 h before I/R surgery); J) Diagram of the experimental protocols for K–M, Atglistatin or an equal volume of vehicle solution was given to mice (50 mg kg^−1^) by *i.p*. injection 1 min post the initiation of reperfusion; K‐M) Plasma CK and LDH levels (K, n = 6 each group), quantification of cardiac infarct size (L, n = 6 each group), and quantification TUNEL‐staining of the ischemic area (M, n = 4 each group) in the hearts of WT mice with or without I/R injury (30‐min ischemia followed by 24‐h reperfusion) with vehicle or Atglistatin treatment (50 mg kg^−1^, *i.p*., 1 min post the initiation of reperfusion). Veh, vehicle; ATGLi, Atglistatin. Data are expressed as Mean ± SEM. Statistical analysis was performed by Student's (F, L and M), one‐way *ANOVA* (H, I and K) and two‐way *ANOVA* (B and C) followed by Tukey's post‐hoc tests. *P < 0.05, **P < 0.01.

To further explore whether the increase in LDs attenuated myocardial I/R injury, we utilized the adipose triglyceride lipase (ATGL) inhibitor Atglistatin, a small molecule that increases LD levels without affecting whole‐body lipid levels. ATGL is a TAG‐decomposing enzyme on the surface of LDs.^[^ ^50^ ^]^ Atglistatin inhibits the decomposition of TAG and increases LD levels.^[^ ^51^ ^]^ Genetic or pharmacological blockade of ATGL reverses the loss of adipose tissue in Seipin KO mice.^[^ ^52^, ^53^ ^]^ Seipin overexpression and Atglistatin treatment had similar effects on cardiomyocyte lipid metabolism during cardiac I/R injury (Figure S6A, Supporting Information). Treatment of cultured NRVMs with Atglistatin markedly elevated the intracellular LD levels (**Figure** **7**A) and alleviated H/R‐induced oxidative stress, inflammation (Figure S6B,C, Supporting Information), and cardiomyocyte death (Figure 7B,C).

This result was confirmed in vivo using a mouse model. Atglistatin treatment before I/R markedly increased LD levels in mouse hearts (Figure 7D,E) and consequently ameliorated myocardial I/R injury (Figure 7F), including I/R‐induced cardiomyocyte death (Figure 7G,H) and cardiac lipotoxicity (Figure 7I; Figure S6D, Supporting Information). Furthermore, administration of Atglistatin 1 min post‐reperfusion also markedly attenuated myocardial I/R injury (Figure 7J–M), as evidenced by reduced levels of plasma markers of cardiomyocyte death (LDH and CK, Figure 7K), the myocardial infarction size (Figure 7L) and apoptosis (indexed by TUNEL staining, Figure 7M ). These data suggested that pharmacological increase of LD levels protected the hearts against I/R injury.

### 2.8 The decrease of Seipin During Acute I/R Injury was Due to the Ischemic Metabolic Adaptation of the Cardiomyocytes

**Figure 8:**
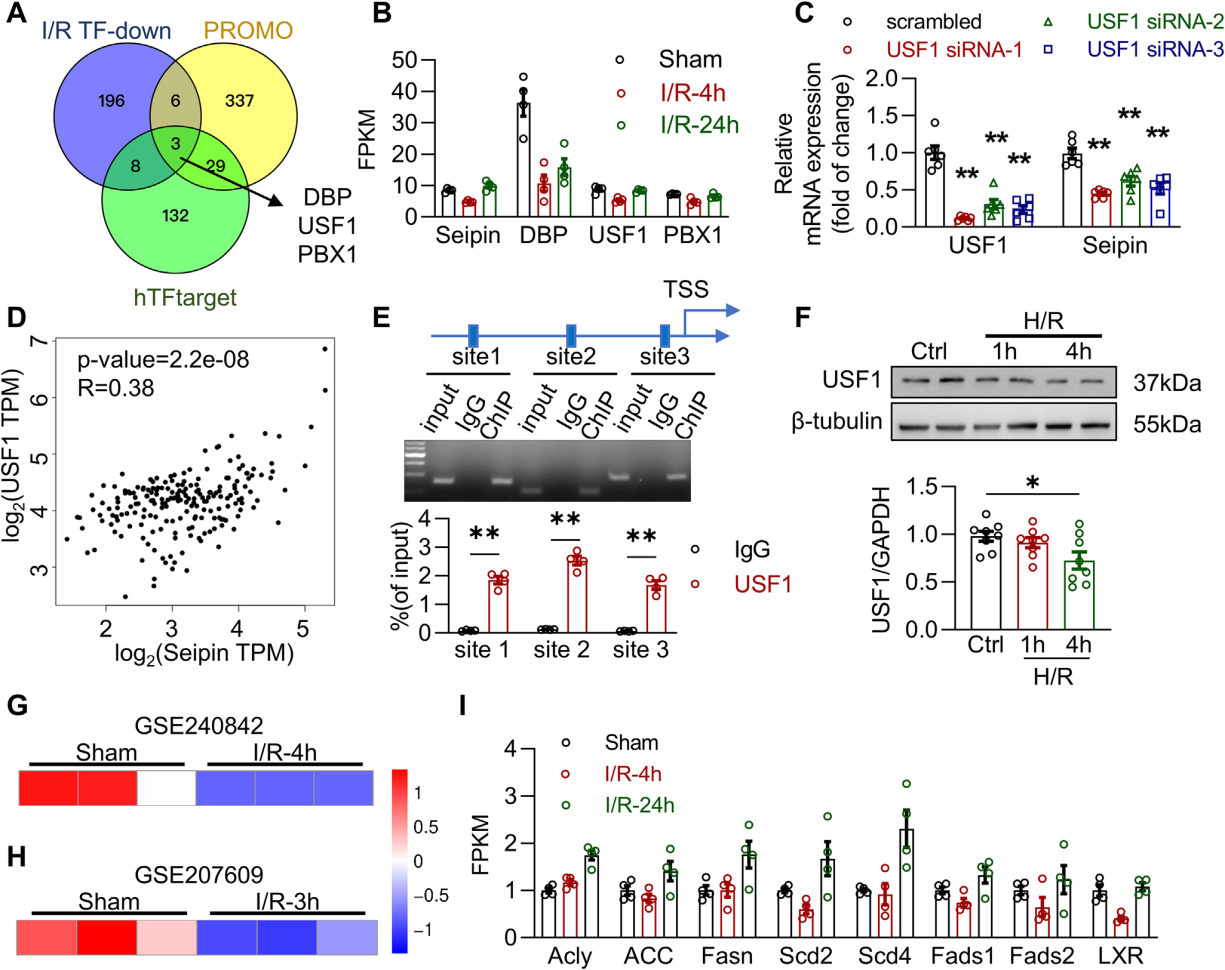
The decrease of Seipin during acute I/R injury is due to the ischemic metabolic adaptation of cardiomyocytes. A) Venn diagram showing transcription factors identified with three databases, including DBP, PBX1 and USF1; B) The FPKM values of Seipin, DBP, PBX1 and USF1 in the myocardial ischemic region by transcriptome sequencing in WT mice with or without I/R injury (30‐min ischemia followed by 4‐h or 24‐h reperfusion; n = 4 each group); C) The mRNA levels of USF1 and Seipin in NRVMs after treatment with scrambled or USF1 siRNA (*n* = 6 each group); D) Correlation between the mRNA levels of USF1 and Seipin from *GEPIA* database. Correlation coefficients are reported as Spearman's rank correlation coefficient; E) ChIP‐qPCR of USF1 and the promoter of Seipin of mice hearts in the bottom with agarose gel on the top (*n* = 4 each group); F) Representative western blotting images and quantitative analysis of USF1 in NRVMs with or without H/R insults (6‐h hypoxia followed by 1‐h and 4‐h reoxygenation, n = 8 each group); G) The mRNA levels of USF1 in WT mice subjected to I/R injury (30‐min ischemia followed by 4‐h reperfusion, n = 3 each group) based on a public data set (GSE240842); H) The mRNA levels of USF1 in WT mice subjected to I/R injury (30‐min ischemia followed by 3‐h reperfusion, n = 3 each group) based on a public data set (GSE207609); I) The FPKM values of genes related to lipogenesis in the myocardial ischemia region by transcriptome sequencing in WT mice with or without I/R injury (30‐min ischemia followed by 4‐h and 24‐h reperfusion, n = 4 each group). TSS, transcription start site. Data are expressed as Mean ± SEM. Statistical analysis was performed by one‐way *ANOVA* (F) and two‐way *ANOVA* (C and E) followed by Tukey's post‐hoc tests. *P < 0.05, **P < 0.01.

We next intend to elucidate why acute I/R injury elevates the reliance on Seipin‐dependent LD formation to store toxic lipids and prevent lipotoxicity, yet suppresses Seipin expression, a contradiction central to lipotoxicity development. During I/R injury (4‐h reperfusion), the mRNA levels of Seipin were downregulated, and we aimed to identify the major transcription factors (TFs) responsible for this downregulation. Based on PROMO and hTFtarget databases, 375 and 172 TFs were predicted to regulate Seipin expression, respectively. Based on RNA‐seq transcriptomic data from our lab,^[^ ^36^ ^]^ 213 TFs were downregulated by I/R injury (4‐h reperfusion) in the heart, which is similar to that of Seipin. Three TFs, D Site‐Binding protein (DBP), USF1, and PBX Homeobox 1 (PBX1), were identified in all three sets of genes and were selected for further analysis (**Figure** **8**A,B).

In cultured NRVMs, USF1 knockdown inhibited Seipin expression (Figure 8C). The positive correlation between myocardial USF1 and Seipin mRNA levels was further confirmed using data from *GEPIA* database (Figure 8D).^[^ ^54^ ^]^ However, the knockdown of DBP or PBX1 had no significant effect on Seipin expression (Figure S7A,B, Supporting Information), and no significant correlation was identified between DBP or PBX1 with Seipin based on data from *GEPIA* database (Figure S7C,D, Supporting Information).

To directly address the mechanistic link between USF1 and Seipin promoter, a chromatin immunoprecipitation (ChIP) assay was performed on mouse heart tissues. Results from the ChIP‐qPCR experiment indicated that USF1 binds to the promoter region of Seipin (Figure 8E). Furthermore, we consulted a publicly available ChIP‐seq dataset from the *ChIP‐Atlas* database, which confirmed the binding of USF1 to Seipin promoter region, which was consistent with our experimental findings (Figure S7E, Supporting Information). Downregulation of USF1 in hearts with acute I/R injury was confirmed by western blotting (Figure 8F) and two different datasets from the GEO database (Figure 8G,H). Therefore, USF1 might be responsible for decreased Seipin expression in cardiomyocytes during I/R.

USF1 is a ubiquitously expressed that TF regulates the transcription of many genes involved in lipid and glucose metabolism pathways.^[^ ^55^ ^]^ USF1 forms a homodimer or heterodimer,^[^ ^56^ ^]^ which binds to the E‐box and activates lipogenic genes.^[^ ^57^ ^]^ Cells exposed to hypoxia convert glucose to lactate, directing carbon away from OXPHOS and lipogenesis.^[^ ^58^, ^59^, ^60^ ^]^ Therefore, a decrease in USF1 levels may be an adaptation of the cardiomyocytes to oxygen deficiency, which could reduce lipogenesis,^[^ ^61^ ^]^ inflammation,^[^ ^62^ ^]^ and cell death.^[^ ^63^ ^]^ We found that the expression of lipogenesis‐related genes markedly decreased during acute ischemia and early reperfusion (4 h) and recovered after 24 h of reperfusion (Figure 8I), which was consistent with the change in USF1 mRNA expression along with its downstream genes (Figure S7F, Supporting Information). Therefore, the downregulation of Seipin and subsequent impairment of LD formation during acute I/R may be side effects of the ischemia‐induced decline in USF1, a master regulator of the genes responsible for lipogenesis.

## 3 Discussion

Here, we found that LD accumulation was upregulated in the heart after I/R injury, although it was insufficient to neutralize lipotoxicity and alleviate cardiomyocyte death. Of all the genes responsible for LD formation, Seipin was identified as an essential LDAP in hearts subjected to I/R injury, and its downregulation was responsible for the insufficient enhancement of LDs in ischemic cardiomyocytes. Increasing LDs levels through Seipin overexpression or the inhibition of lipolysis protected the heart from I/R injury. The insufficient upregulation of Seipin was attributable to a decrease in its transcription factor USF1, which is essential for the metabolic adaptation of cardiomyocytes during myocardial ischemia (see Graphical Abstract). Our study revealed the role of LDs in cardiac I/R injury and the pivotal role of Seipin‐mediated LD biogenesis in IHD.

During cardiac I/R injury, reperfusion initiates oxidative stress, which leads to the release of lipid radicals from membrane TAGs, phospholipids, and chain damage, leading to cell death.^[^ ^48^, ^64^ ^]^ The critical event in this series of harmful events is the unwanted accumulation of FFA and detrimental metabolites. Abnormal accumulation of toxic lipids (FFA, DAG, ceramide, etc.) leads to lipotoxicity, which elicits inflammation, oxidative stress, and other pathological processes, and plays an important role in I/R‐induced cardiomyocyte injury and death.^[^ ^20^ ^]^ Ceramide accumulation is a well‐documented driver of cardiomyocyte apoptosis. Ceramides directly induce mitochondrial outer membrane permeabilization by facilitating Bax activation and inhibiting anti‐apoptotic Bcl2 proteins, ultimately triggering caspase activation and cell death.^[^ ^65^ ^]^ Concurrently, elevated levels of FFAs, particularly saturated FFAs such as palmitate, activate pro‐inflammatory and pro‐apoptotic cascades in cardiomyocytes.^[^ ^66^ ^]^

LDs are key organelles in lipid metabolism and energy homeostasis and are involved in various diseases, including obesity, lipodystrophy, cancer, and cardiovascular diseases.^[^ ^30^ ^]^ However, to date, a consensus on the effects and mechanisms of LDs on I/R injury has not been established. It has been reported that LDs could sequester toxic lipid intermediates and protect the heart from lipotoxicity.^[^ ^67^, ^68^ ^]^ However, in this study, myocardial LDs increased after I/R injury. Further enhancement of the level of LDs genetically or pharmacologically, significantly reduced the accumulation of toxic lipids, thereby alleviating lipotoxicity, cardiomyocyte damage, and death in hearts subjected to I/R injury. Thus, although LDs were upregulated during I/R injury, they were insufficient to counteract lipotoxicity and cardiomyocyte damage. Therefore, upregulating LD represents an important target for preventing and mitigating cardiac I/R injury. The protective effects of Seipin overexpression and Atglistatin treatment may be critically dependent on basal lipid homeostasis. Under normolipidemic conditions, these interventions enhance LD biogenesis and storage capacity, thereby sequestering excess fatty acids and preventing lipotoxicity. The activation of liver X receptors (LXR) in the heart leads to the accumulation of LDs and attenuation of I/R injury,^[^ ^33^ ^]^ and inhibiting LD breakdown could alleviate ferroptosis and attenuate I/R injury in mice.^[^ ^32^ ^]^ However, other studies have proposed that excessive and prolonged accumulation of LDs in the heart, in the context of pre‐existing hyperlipidemia or excessive lipid supply, together with the liver and kidney,^[^ ^69^, ^70^ ^]^ is associated with multiple disorders during obesity^[^ ^71^ ^]^ and diabetes.^[^ ^72^ ^]^ Excess lipid accumulation in the heart is associated with decreased cardiac function in metabolic disorder‐associated heart diseases.^[^ ^73^, ^74^ ^]^ Despite enhanced LD formation, severe lipid overload exceeds the protective threshold, leading to dysregulated lipolysis, excessive lipid peroxidation, and amplified inflammatory signaling, ultimately aggravating I/R injury.^[^ ^75^ ^]^ Therefore, only proper and moderate LD levels can have a protective effect on the heart, whereas excessive LDs levels are harmful to the organs.

The increase in LDs after cardiac I/R injury observed in our study was consistent with other reported studies.^[^ ^32^, ^33^, ^34^ ^]^ However, these studies have not elucidated the molecular regulatory mechanisms underlying LD biogenesis during cardiac I/R injury. The biogenesis of LDs is a complex process. The first step is the synthesis of neutral lipids catalyzed by different enzymes.^[^ ^31^, ^76^ ^]^ Seipin and other LD biogenesis factors are then recruited to facilitate the growth of nascent LDs. Finally, LDs bud from the ER and grow via fusion or local lipid synthesis. Here, based on a transcriptome database combined with subsequent experimental validation in vivo and in vitro, we demonstrated that Seipin plays a central role in the regulation of LD biogenesis in cardiomyocytes during I/R injury. RNA‐seq transcriptome data showed that four LDAPs were significantly altered in heart tissues subjected to acute I/R injury. However, the knockdown of Seipin resulted in an abnormal reduction in LDs. Cardiac LDAP dysfunction may affect cardiac function and result in cardiomyopathy. ATGL inhibition by Atglistatin can alleviate ischemic brain injury and myocardial remodeling.^[^ ^77^, ^78^ ^]^ Plin5 is highly abundant in the heart and protects it against excessive oxidative stress. Plin5 deficiency exacerbates pressure overload‐induced cardiac hypertrophy and heart failure,^[^ ^79^ ^]^ and cardiac‐specific overexpression of Plin5 protects against lipotoxicity‐induced heart dysfunction in metabolic disease.^[^ ^80^ ^]^ However, the deletion of Plin5 did not influence ischemic damage in isolated, perfused mouse hearts or cardiomyocytes,^[^ ^81^ ^]^ which is consistent with our current study. ABHD5 protects against pressure overload‐induced heart failure,^[^ ^82^ ^]^ although ABHD5 was not changed in I/R injury. Although different LDAPs regulate the formation and function of LDs in different disease processes, Seipin is a major regulator of LD formation in myocardial I/R injury.

During acute myocardial I/R injury, Seipin expression is decreased despite metabolic abnormalities and increased demand for LD in cardiomyocytes. Although based on the compensatory response of cardiomyocytes, the LD content of cardiomyocytes still increased after I/R injury, even with a decline in Seipin, it could not meet the demands of cardiomyocytes, resulting in lipotoxicity and cell death. Seipin overexpression promoted LD formation and further increased LD levels both in mouse hearts and cultured cardiomyocytes, alleviating lipotoxicity and cardiomyocyte death in cardiac I/R (H/R) injury. Seipin deficiency inhibited LD formation and aggravated cardiac I/R (H/R) injury. Therefore, improving LD levels during cardiac I/R by increasing Seipin levels or other methods is an important treatment strategy for acute cardiac I/R injury.

Regarding the mechanisms of the divergence between increased LD demand and decreased Seipin expression, the downregulation of Seipin may be an adverse consequence of the ischemia‐induced decline in USF1, a transcription factor identified in our study to regulate the expression of Seipin. The delivery and utilization of O_2_ are important for cell survival. When myocardial ischemia occurs because of a lack of oxygen and various nutrients, metabolic adaptation occurs in cardiomyocytes to maintain cell survival.^[^ ^83^ ^]^ Hypoxia significantly decreases intracellular *de novo* lipogenesis,^[^ ^84^, ^85^ ^]^ as it is a costly process that requires AcCoA, NADPH, and ATP to synthesize one palmitate molecule.^[^ ^86^ ^]^ Lipogenesis decreases by 43%, and spontaneous lipolysis increases by 135% under severe hypoxia.^[^ ^60^ ^]^ USF1 and LXR are both the critical transcriptional regulators of lipogenesis,^[^ ^55^ ^]^ and both USF1 and LXR decreased significantly in acute I/R. The expression of genes involved in lipogenesis was also markedly decreased during acute ischemia. These metabolic changes would decrease lipogenesis and unnecessary energy expenditure, thereby improving cardiomyocyte metabolism under hypoxic conditions. However, because USF1 is an important transcription factor for Seipin, a decrease in USF1 also decreased Seipin. We also discovered for the first time the transcriptional regulatory effect of USF1 on Seipin. During acute reperfusion (4 h), USF1 cannot return to normal levels in a short period of time, resulting in a deficient amount of Seipin in cardiomyocytes. This leads to an insufficient generation of LDs during acute I/R injury, which constitutes an important mechanism of I/R‐induced lipotoxicity and cardiomyocyte damage.

In mature adipocytes and testes cells, Seipin‐deficient cells have more but smaller LDs, accompanied by a few very large LDs.^[^ ^87^, ^88^, ^89^, ^90^ ^]^ In this study, Seipin‐KO cardiomyocytes contained very few LDs. The presence of large LDs was not observed, possibly because cardiac tissue has a relatively lower lipid content than adipose and testicular tissues. Seipin deficiency mainly manifests as systemic fat loss, fatty liver, insulin resistance, and other metabolic diseases, accompanied by hypertrophic cardiomyopathy and kidney damage.^[^ ^91^ ^]^ The occurrence of cardiomyopathy is mainly related to disorders of glucose and lipid metabolism.^[^ ^47^, ^53^, ^92^ ^]^ Similarly, in this study, based on lipidomic data, Seipin KO increased the levels of toxic lipids, including ceramide and FFA, in the heart, which is consistent with published data.^[^ ^49^ ^]^ Several studies have shown that Seipin overexpression has protective effects on multiple organs. Seipin overexpression alleviates hepatic steatosis by influencing intracellular calcium level^[^ ^93^ ^]^ and attenuates cerebral I/R injury by preventing apoptosis and autophagy.^[^ ^94^ ^]^ We confirmed that Seipin alleviated lipotoxicity by promoting LD formation, which ultimately reduced myocardial I/R injury. Therefore, we cannot exclude the possibility that other functions of Seipin are involved in the regulation of acute cardiac I/R injury.

Our study had some limitations. This study was conducted using a rodent model. Further experiments using human cardiomyocytes and cardiac samples are required to validate the clinical significance of our findings. In addition, LDs were involved in the regulation of calcium signaling.^[^ ^95^, ^96^ ^]^ Recent studies have reported that Seipin regulates Serca2A, an important ER calcium pump, under physiological and pathological conditions.^[^ ^97^, ^98^ ^]^ Considering the critical role of calcium signaling in initiating I/R injury,^[^ ^59^ ^]^ a more complex network may exist between Seipin, calcium flux, and LDs in cardiomyocytes. However, further research is required to confirm this hypothesis.

In summary, we identified the protective effects of LDs and Seipin against myocardial I/R injury. We demonstrate that Seipin‐mediated LD formation serves as a protective mechanism against I/R‐induced lipotoxicity by sequestering toxic lipid intermediates. Enhancing LDs levels through either Seipin overexpression or pharmacological inhibition of lipolysis effectively ameliorates myocardial I/R injury by reducing lipotoxicity and cell death. We not only demonstrate that Seipin and LDs play an essential role in maintaining cardiac lipid homeostasis during ischemic stress, but also identify the Seipin‐LD axis as a promising therapeutic target for the prevention and therapy of myocardial I/R injury and its complications.

## 4 Experimental Section

### Animal Care and Use

All animal experiments were approved by the Institutional Animal Care Research Advisory Committee of Peking University Health Science Center (LA2020509) and the Guide for the Care and Use of Laboratory Animals (8^th^ edition, The National Academies Press, 2011) of the Association for Assessment and Accreditation of Laboratory Animal Care. Male C57BL/6J mice were provided by the Animal Center of Peking University Health Science Center. All mice were maintained under controlled environmental conditions of temperature (22 °C ± 0.5 °C), humidity (60% ± 5%), and lighting (12‐h light/12‐h dark cycle) and provided ad libitum access to standard chow and water. The animals were randomly allocated to experimental groups.

To investigate the role of Seipin in myocardial I/R injury, male WT mice, Seipin KO mice and cardiomyocyte‐specific Seipin‐overexpressing C57BL/6J mice were used to investigate the role of Seipin in myocardial I/R injury. Seipin KO mice were generated and bred as previously described,^[^ ^45^, ^46^ ^]^ and WT littermates were used as controls.

To establish cardiomyocyte‐specific Seipin‐overexpressing mice, AAV9 carrying cTnT promoter‐driven Seipin‐3xFlag (AAV9‐Seipin) or an empty vector (AAV9‐Ctrl) was constructed by Obio Technology Co., Ltd., China. The 6‐week‐old male WT mice were randomized into two different groups, which were injected with AAV9‐Seipin (defined as AAV‐Seipin mice, 2 × 10^10^ vg/g body weight) or AAV9‐Ctrl (AAV‐Ctrl mice, 2 × 10^10^ vg/g body weight) via the tail vein 4 weeks before I/R surgery or a sham operation.

To establish cardiac‐specific Seipin‐knockdown mice, AAV9 vectors carrying cTnT promoter‐driven shRNA targeting Seipin (AAV‐shSeipin) and scrambled control shRNA (AAV‐shNC) were constructed by Hanbio Tech (Shanghai, China). The 6‐week‐old male WT mice were randomized into two different groups, which were injected with AAV‐shSeipin (defined as AAV‐shSeipin mice, 2 × 10^10^ vg/g body weight) or AAV‐shNC (defined as AAV‐shNC mice, 2 × 10^10^ vg/g body weight) via the tail vein. Subsequent experiments were performed 4 weeks post‐injection to allow for efficient transduction and gene knockdown. Two specific shRNA sequences targeting Seipin were used: sequence 1, GGCUCCUUCUACUACUCCU and sequence 2, GCAGGUUAAUAUCCGACAA.

To explore the effect of increasing LDs levels on I/R injury, Atglistatin (HY‐15859, MCE, USA) or an equal volume of vehicle solution was administered to 10‐week‐old WT mice (50 mg kg^−1^) randomly by intraperitoneal (*i.p*.) injection 4 h before I/R surgery, 1 min post the initiation of reperfusion, or sham operation.^[^ ^77^ ^]^

### Myocardial I/R Model in Mice

According to previous protocols,^[^ ^36^ ^]^ 10‐week‐old mice were anesthetized with sodium pentobarbital (60 mg kg^−1^) via *i.p*., and a rodent respirator (ALCV9A, Shanghai Alcott Biotech) was used for ventilation. Following a skin incision, the heart was exposed by left thoracotomy through the 3^rd^ or 4^th^ intercostal space. After the pericardium was removed, the left anterior descending coronary artery (LAD) was occluded with a 6–0 silk suture for 30 min. Effective ligation of the LAD was confirmed by myocardial blanching, ventricular dyskinesia, and elevation of the ST segment on the ECG. The sham‐operated mice were subjected to a procedure without LAD ligation. After 4 hours, 24 hours, or 3 weeks of reperfusion, the mice were sacrificed to collect blood samples for LDH and CK measurements. Heart samples were isolated from ischemic areas of the mice for further analysis.

### I/R Injury Assessment

TTC staining was performed as a previous protocol.^[^ ^37^ ^]^ After reperfusion (24 h), we ligated the LAD again and retrogradely infused the heart with 0.05% Evans blue from the aorta. The non‐ischemic part of the ventricle was stained blue, and the non‐stained part represented the area at risk (AAR). The heart was then frozen for 15 min at −80 °C and segmented into six slices. The heart slices were incubated in 1% TTC (8877, Sigma, USA) in phosphate buffer (pH 7.4) for 10 min at 37 °C and then immersed in 4% paraformaldehyde for 24 h. Infarct size (IS, white region) was differentiated from non‐infarcted AAR (red region) by TTC staining. Finally, the slices were arranged from base to apex and photographed digitally. Digital images of the heart slices were analyzed using the ImageJ software to measure the IS and AAR regions. The results are presented as IS/AAR% for in vivo experiments. The data were analyzed blindly.

For plasma LDH and CK, blood samples were collected by retro‐orbital puncture with a heparinized capillary tube after 4 h reperfusion, and anticoagulated blood was centrifuged (4000 rpm, 4 °C, 10 min) to separate the plasma. LDH was spectrophotometrically measured using an LDH assay kit (MAK066, Sigma, USA) following the manufacturer's instructions. CK was measured spectrophotometrically using a CK assay kit (CPK0300, Jingyuan, China) following the manufacturer's instructions.^[^ ^37^ ^]^ The LDH and CK values were assessed seven times at a wavelength of 340 nm.

### Statistics and Reproducibility

All the experiments and data analyses were performed in a blinded manner. All data are presented as means ± standard errors of the mean (SEM). Normality was assessed using the Shapiro‐Wilk test, and homogeneity of variances was verified using the Brown‐Forsythe test. For normally distributed data, a two‐sided unpaired Student's *t*‐test was used to detect significant differences between two groups. A one‐way or two‐way analysis of variance (*ANOVA*) was performed to compare differences among three or more groups. Tukey's post‐hoc test was used for all *ANOVA*s, as recommended. Otherwise, the differences were analyzed using a nonparametric test (Mann–Whitney *U* test) between the two groups and Kruskal‐Wallis test among three or more groups. Dunn's post‐hoc test was used for Kruskal‐Wallis test. The sample size (n) represents the number of biologically independent animals (or cell culture preparations). GraphPad Prism for Windows (version 9.5.1) was used for the statistical analyses. No statistical methods were used to predetermine the sample size. Statistical significance was set at *P* < 0.05.

## Conflict of Interest

The authors declare no conflict of interest.

## Author Contributions

Y.Z. and W.H. conceived and designed the study. C.L. performed most of the experiments. Y.C., G.S., Z.Z., Y.H., J.C., and Y.L. provided assistance in some experiments. H.S. provided assistance in animal experiments. J.Z., X.K., H.Q., and J.D. contributed to the discussion and provided reagents. Y.Z., W.H., and J.Z. supervised the study. Y.Z., W.H., and C.L. analyzed the data. Y.Z., W.H., C.L., and J.Z. wrote the paper. All authors have read and approved the final manuscript.

## Supporting information



Supporting Information

Supporting Information

## Data Availability

The data that support the findings of this study are available from the corresponding author upon reasonable request.
