# Supplementary material for: Seipin‐Mediated Lipid Droplet Formation in Cardiomyocytes Ameliorates Cardiac Ischemia/Reperfusion Injury
Source: Adv Sci (Weinh). 2025 Nov 19;13(6):e10203. doi: 10.1002/advs.202510203 (PMC12866716; doi:10.1002/advs.202510203)
Supplement: Supplementary file 1 — Supporting Information [file ADVS-13-e10203-s002.pdf]

Supporting Information

Seipin-mediated Lipid Droplet Formation in Cardiomyocytes Ameliorates Cardiac Ischemia/reperfusion Injury

Changyun Liu, Junxia Zhang, Yusi Chen, Geng Shen, Yufei Han, Zihao Zhou, Jinxuan Chen, Xuya Kang, Huilin Qu, Jiaxin Duanmu, Haibao Shang, Yingjia Li, Wei Huang, Yan Zhang**

Supplemental Figures

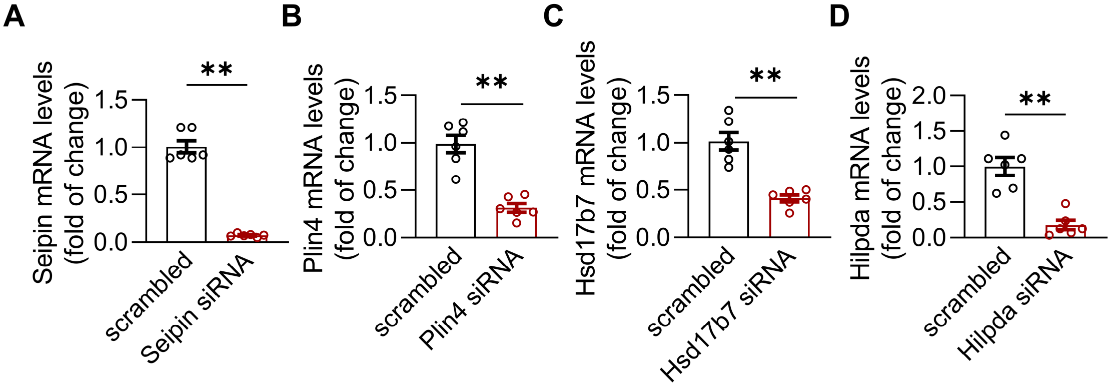

Supplemental Figure S1: Verification of the gene knockdown by siRNAs.

Quantitative analysis of Seipin (**A**), Plin4 (**B**), Hsd17b7 (**C**) and Hilpda (**D**) mRNA levels by qPCR in NRVMs transfected with scrambled or siRNAs of LD-associated proteins, n=6 each group. Data are expressed as Mean ± SEM. Statistical analysis was performed by Student's *t* test (**B, C** and **D**) and Mann–Whitney *U* test (**A**). Differences are significant for *$P<0.05$, **$P<0.01$.

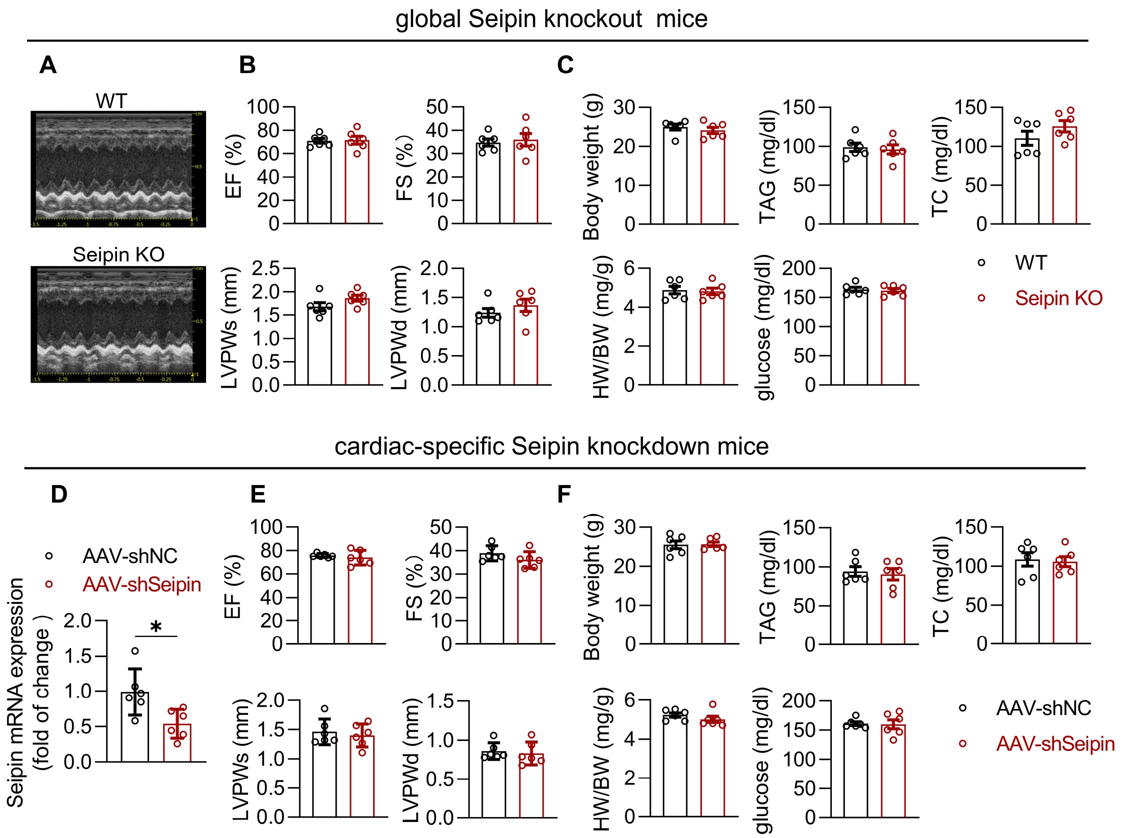

Supplemental Figure S2: Knockout of Seipin did not alter the systematic metabolism and heart function under normal condition.

A-C) Representative M-mode echocardiography images (**A**) and quantitative analysis (**B**), quantitative analysis of body weight, heart weight/body weight (HW/BW), 4-h fasting plasma glucose, TC and TAG (**C**) of 10-week-old WT and Seipin KO mice, n=6 each group; **D-F)** The mRNA level of Seipin in the hearts (**D**), quantitative analysis of M-mode echocardiography (**E**), quantitative analysis of body weight, heart weight/body weight (HW/BW), 4-h fasting plasma glucose, TC and TAG (**F**) of AAV-shNC and AAV-shSeipin mice, n=6 each group. NC, negative control. Data are expressed as Mean ± SEM. Statistical analysis was performed by Student's *t* test. Differences are significant for **P*<0.05.

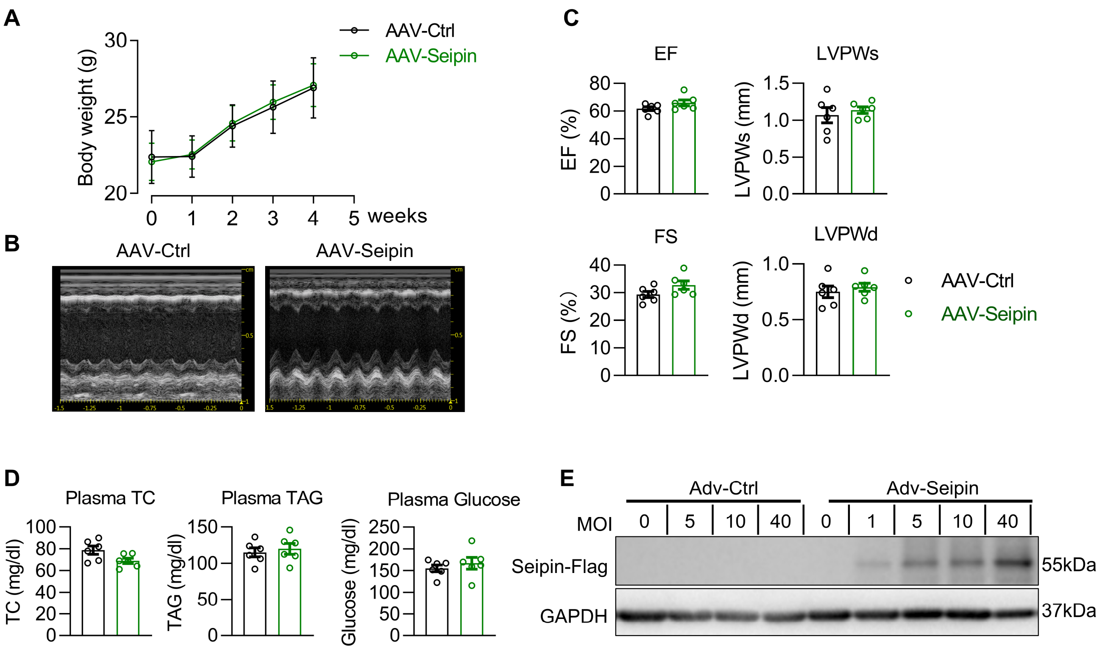

Supplemental Figure S3: Cardiomyocyte-specific overexpression of Seipin did not alter the systematic metabolism and heart function under normal condition.

A) Quantitative analysis of body weight of the WT mice every week after injected with AAV-Ctrl or AAV-Seipin via the tail vein (n=12 each group); **B-C**) Representative M-mode echocardiography images (**B**) and quantitative analysis (**C**) of the hearts of WT mice 4 weeks after injected with AAV-Ctrl or AAV-Seipin via the tail vein, n=6 each group; **D**) Quantitative analysis of 4-h fasting plasma TC, TAG, and glucose of WT mice 4 weeks after injected with AAV-Ctrl or AAV-Seipin via the tail vein (n=6 each group). **E**) Representative images of western blots of Seipin-Flag protein level of NRVMs infected with Adv-Ctrl or Adv-Seipin at different MOI for 48 hours. MOI, multiplicity of infection. Data are expressed as Mean ± SEM. Statistical analysis was performed by Student's *t* test.

Supplement figure 4

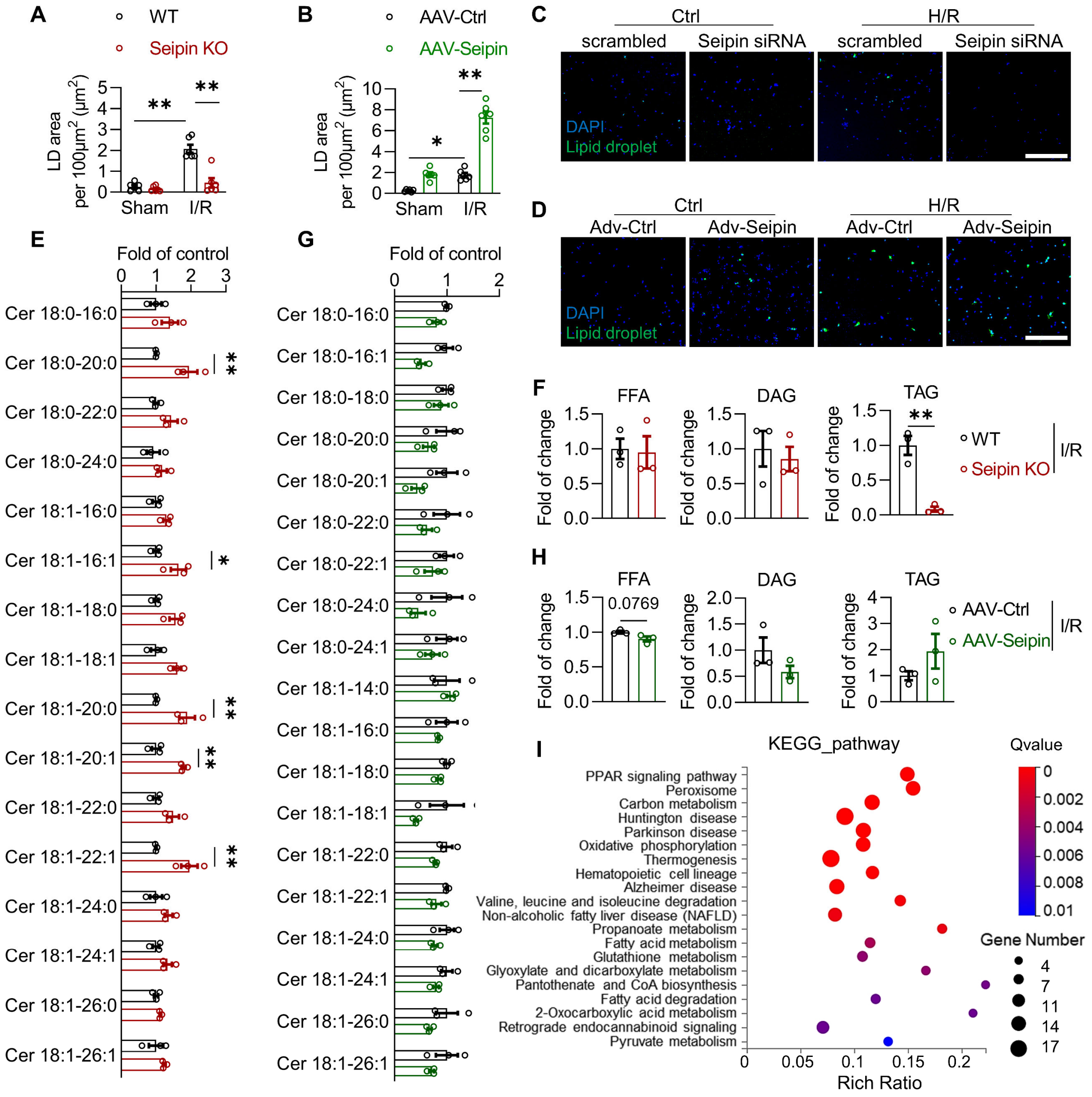

Supplemental Figure S4: Seipin promotes LDs formation and improve lipid metabolism in the hearts with I/R injury.

A-B) Quantitative analysis of LDs area per 100 μm^2 of fluorescence images in myocardium of WT and Seipin KO mice (**A**), AAV-Ctrl and AAV-Seipin mice (**B**) with or without I/R injury (30-min ischemia followed by 24-h reperfusion, n=6 each group); **C-D**) Representative fluorescence images indicating LDs labeled with Bodipy 493/503 and nucleus labeled with DAPI of NRVMs transfected with scrambled/Seipin siRNA (**C**) or Adv-Ctrl/Adv-Seipin (**D**) with or without H/R insults (6-h hypoxia followed by 4-h reoxygenation; scale bar, 200 μm); **E-F**) Quantitative analysis of ceramide (**E**), total FFA, DAG and TAG (**F**) based on lipidomic

analysis of myocardium of WT and Seipin KO mice with I/R injury (30-min ischemia followed by 24-h reperfusion; n=3 each group); **G-H**) Quantitative analysis of ceramide (**G**), total FFA, DAG and TAG (**H**) based on lipidomic analysis of myocardium of AAV-Ctrl and AAV-Seipin mice with I/R injury (30-min ischemia followed by 24-h reperfusion; n=3 each group); **I**) KEGG pathway enrichment of RNA-seq data in the hearts of WT and Seipin KO mice with or without I/R injury (30-min ischemia followed by 24-h reperfusion; n=3 for sham group and n=4 for I/R group). Data are expressed as Mean ± SEM. Statistical analysis was performed by Student's *t* test (**F** and **H**) and two-way *ANOVA* (**A, B, E** and **G**) followed by Tukey’s post-hoc tests. *P<0.05, **P<0.01.

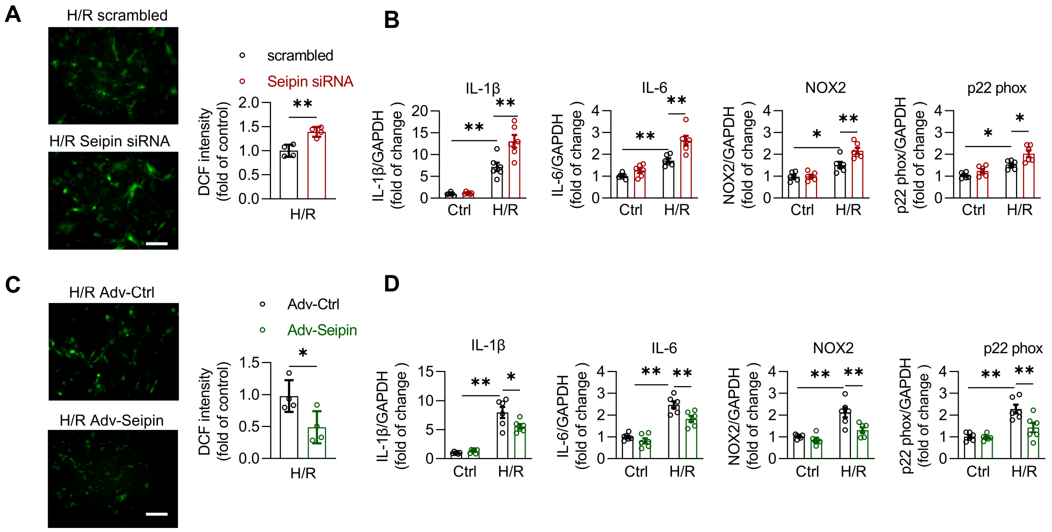

Supplemental Figure S5: Seipin is essential for lipotoxicity-elicited cardiac pathology induced by H/R injury.

A) Representative fluorescence images and averaged data for ROS production (as assessed by DCF fluorescence intensity) of NRVMs transfected with scrambled or Seipin siRNA with H/R insults (6-h hypoxia followed by 4-h reoxygenation; n=4 each group). The DCF-stained ROS are shown in green (scale bar, 100 μm); **B**) The mRNA levels of IL-1β, IL-6, NOX2 and p22 phox were detected using qPCR in NRVMs transfected with scrambled or Seipin siRNA with or without H/R insults (n=6 each group); **C**) Representative fluorescence images and averaged data for ROS production (as assessed by DCF fluorescence intensity) of NRVMs infected with Adv-Ctrl or Adv-Seipin (MOI 10, 48h) with H/R insults (6-h hypoxia followed by 4-h reoxygenation; n=4 each group). The DCF-stained ROS are shown in green (scale bar, 100 μm); **D**) The mRNA levels of IL-1β, IL-6, NOX2 and p22 phox were detected using qPCR in NRVMs infected with Adv-Ctrl or Adv-Seipin (MOI 10, 48h) with H/R insults (6-h hypoxia followed by 4-h reoxygenation; n=6 each group). MOI, multiplicity of infection. Data are expressed as Mean ± SEM. Statistical analysis was performed by Student's t test (**A** and **C**) and two-way *ANOVA* (**B** and **D**) followed by Tukey's post-hoc tests. *$P<0.05$, **$P<0.01$.

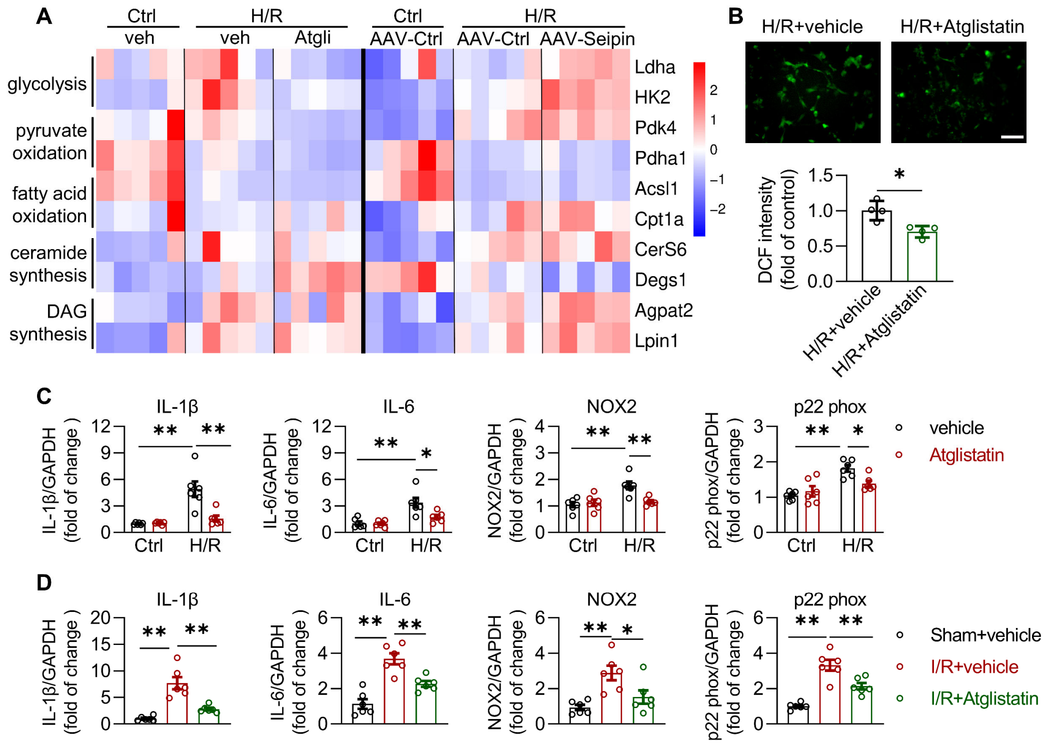

Supplemental Figure S6: Upregulation of LD levels alleviated H/R injury in NRVMs.

A) The mRNA levels of Ldha, HK2, Pdk4, Pdha1, Acsl1, Cpt1a, CerS6, Degs1, Agpat2 and Lpin1 of NRVM treated with vehicle/Atglistatin or Adv-Ctrl/Adv-Seipin with or without H/R insults (6-h hypoxia followed by 4-h reoxygenation; n=5). 10 µM Atglistatin or equal vehicle was added to the medium 1 hour before hypoxia. Adv-Ctrl or Adv-Seipin was added to medium at 10 MOI for 48 hours; **B**) Representative fluorescence images and averaged data for ROS production (as assessed by DCF fluorescence intensity) of NRVMs subjected to H/R (6-h hypoxia followed by 4-h reoxygenation) with vehicle or Atglistatin treatment (n=4 each group). 10µM Atglistatin was added to the medium 1 hour before hypoxia. The DCF-stained ROS are shown in green; **C**) The mRNA levels of IL-1β, IL-6, NOX2 and p22 phox were detected by qPCR in NRVMs subjected to H/R with vehicle or Atglistatin treatment. 10µM Atglistatin was added to the medium 1 hour before hypoxia; **D**) The mRNA levels of IL-1β, IL-6, NOX2 and p22 phox were detected using qPCR in the hearts of WT mice subjected to I/R injury (30-min ischemia followed by 24-h reperfusion) with vehicle or Atglistatin treatment (50 mg/kg, *i.p.*, 4 hours before I/R surgery, n=6 each group). Data are expressed as Mean ± SEM. Statistical analysis was performed by Student's t test (**B**), one-way *ANOVA* (**D**) and two-way *ANOVA* (**C**) followed by Tukey's post-hoc tests. Differences are significant for **P*<0.05, ***P*<0.01.

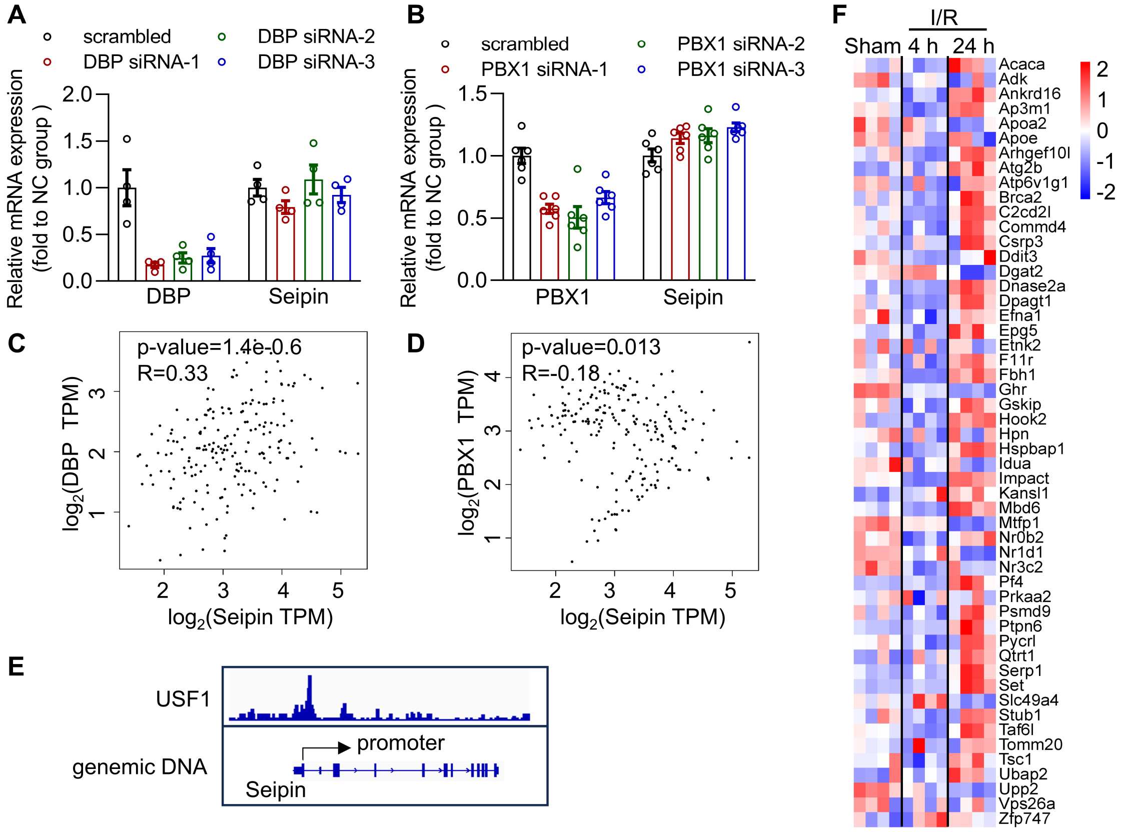

Supplemental Figure S7: DBP and PBX1 do not regulate the transcription of Seipin , while USF1 binds to the Seipin promoter..

A) The levels of DBP and Seipin mRNA were detected using qPCR in NRVMs transfected with scrambled or DBP siRNA for 48h (n=4 each group); **B**) The levels of PBX1 and Seipin mRNA were detected by qPCR in NRVMs transfected with scrambled or PBX1 siRNA for 48h (n=6 each group); **C-D**) Correlation between the mRNA levels of DBP and Seipin (**C**), PBX1 and Seipin (**D**) based on the data from *GEPIA* database, Correlation coefficients are reported as Spearman's rank correlation coefficient; **E**) The *ChIP-Atlas* analysis of enrichment of Seipin promoter; **F**) The FPKM values levels of genes downstream of the USF1 pathway in the myocardial ischemia region by transcriptome sequencing in WT mice with or without I/R injury (30-min ischemia followed by 4-h and 24-h reperfusion, n=4 each group). Data are expressed as Mean ± SEM. Statistical analysis was performed by two-way *ANOVA* (**A** and **B**) followed by Tukey's post-hoc tests. *P<0.05, **P<0.01.

Supplemental Materials & Methods

Assessment of blood lipid and glucose in mice

Plasma samples were collected from the retro-orbital plexus of mice after 4-hour fasting, and plasma was separated from the blood by centrifugation for 10 min at 4000 rpm. The levels of plasma TAG, TC, and glucose were measured using the following kits: Triglyceride Assay Kit (100000220, BioSino), Cholesterol Assay Kit (100000180, BioSino), and Glucose Assay Kit (100000240, BioSino).[1]

Echocardiography

Cardiac function was assessed by echocardiography using a Vinno6VET device (Vinno Technology, China) as previously described.[2] Mice were anesthetized with 3% isoflurane (Baxter Healthcare Corp, New Providence, RI, USA) and maintained asleep with 1.0-1.5% isoflurane to keep the heart rate stabilized at 400-500 beats per minute. At the level of the papillary muscle of the heart in mice, two-dimensional parasternal long-axis views and short-axis views were obtained. The M-mode cursor was positioned perpendicular to the maximum LV dimension during end-diastole and systole. The following variables were measured digitally in the M-mode images: LVPWd (mm), left ventricular posterior wall thickness at diastolic phase; LVPWs (mm), left ventricular posterior wall thickness at systolic phase; LVIDs (mm), left ventricular internal dimension in systole; LVIDd (mm), left ventricular internal dimension in diastole. The ejection fraction (EF) and fractional shortening (FS) were calculated from echocardiography parameters. The researcher who performed echocardiography was blinded to the mouse genotypes.

Histological analysis and tissue immunofluorescence

The ischemic cardiac tissues were taken for immunofluorescence staining. After fixation with 4% paraformaldehyde and dehydration with 30% sucrose solution, the tissues were embedded in OCT and sectioned at a thickness of 7 μm. Subsequently, the slices were blocked with 7.5% normal goat serum for 1 hour, incubated with the VCAM-1 (A01199, Boster, China) antibody at 4°C overnight, and followed by incubation with Alexa Fluor 488-conjugated secondary

antibodies to visualize the staining. Diamidino phenylindole (DAPI) reagent (D9542, Sigma, USA) was used to counterstain the nuclei. The staining was observed, and images were captured using a fluorescence microscope. The data were analyzed blindly.

Masson's trichrome staining, and immunohistochemistry were performed as previously reported.[3] The apoptosis levels of myocardial tissue were detected using a TUNEL kit (11684795910, Roche Applied Science, USA) according to the manufacturer’s instructions.[3] The data were analyzed blindly.

Measurement of cardiac ROS

The ROS levels in the ischemic myocardial area were determined by dihydroethidium (DHE, D11347, Invitrogen, USA) staining. Briefly, transverse cryosections (7 μm thick) of freshly resected tissue were washed with 0.01% PBS three times before incubation of DHE (5 μM) for 30 minutes at room temperature. DAPI reagent was used to counterstain the nuclei. DHE fluorescence was observed and images were captured using fluorescence microscope. The data were analyzed blindly.

Cardiomyocyte isolation, adenoviral infection, administration of Atglistatin, hypoxia/ reoxygenation and ROS measurement

NRVMs were isolated from 1-day-old Sprague-Dawley rats as previously described.[4] The harvested cells were plated on glass coverslips in 12-well plates (Corning, USA) at 1×10^5 cells per well for Bodipy 493/503 and ROS staining.

The adenoviral vector expressing human Seipin was constructed by Obio Technology Co. Ltd., China. For adenovirus transfection, cells were incubated with DMEM (12100046, Gibco, USA) containing adenovirus for 6-8 h. Cells were collected for further analysis 48-72 h after transfection.

To improve cellular LDs levels, NRVMs were pretreated with different concentrations of Atglistatin dissolved in DMSO for 1 hour before H/R. [5]

Cardiomyocyte H/R was performed as previously described. [4] To induce cardiomyocyte hypoxia, NRVMs were cultured in DMEM with 10% fetal bovine serum for 48-72 h. The

medium was then changed to serum-free RPMI 1640 (11879020, Gibco, USA), and the cells were placed in a 37°C airtight chamber saturated with 95% N_2/5% CO_2 for 6h (the O_2 concentration was <0.1% (Type 5120, Ohmeda Oxygen Monitor, USA)). When cardiomyocytes were reoxygenated, the culture medium was changed to DMEM, and the cells were placed in a 37°C/5% CO_2 incubator for 1-4 h before analysis.

After H/R, relative cell viability was detected using a cell counting kit-8 (CCK-8, CA1210, Solarbio, China) according to the manufacturer's instructions, and LDH level in the culture medium were measured to determine the degree of cell damage using a kit from Sigma (MAK066).[3]

To assess ROS production in cultured NRVMs, the cell-permeant indicator 5-(and-6)-chloromethyl-2,7-dichlorodihydrofluorescein diacetate acetyl ester (DCF, CM-H2DCFDA, Invitrogen, USA) was used. After washing with PBS, the NRVMs were incubated with 10 μM DCF for 30 min, allowed to recover for 15 min in full growth medium, and then washed again with PBS. The staining was observed, and images were captured, measured at 483 nm excitation and 520 nm emission using a fluorescence microscope.

Measurement of the cardiac LD content: Bodipy 493/503 staining and quantification

The level of LDs in the heart was determined by Bodipy 493/503 (D3922, Thermo Fisher Scientific, USA). Briefly, transverse cryosections of frozen hearts or fixed NRVMs were incubated with 20 μM Bodipy 493/503 at 37°C for 30 min. DAPI reagent was used to counterstain nuclei. Bodipy 493/503 fluorescence was assessed by fluorescence microscopy. Images were analyzed using ImageJ software to determine the area and number of LDs. The data were analyzed blindly.

For the quantification of LDs in heart tissue sections, the number and total cross-sectional area of LDs were quantified per 100 μm^2 of tissue area. The mean LD area was subsequently calculated by dividing the total LD area by the number of LDs within the same reference area.

For the quantification of LDs in NRVMs, the total LD area per field of view was measured, and the number of nuclei in the same field was counted. The mean LD area per cell was then calculated by dividing the total LD area by the number of nuclei.

Transmission electron microscopy (TEM) and quantification

Cardiac tissues were dissected and poured in the fixative (2.5% glutaraldehyde in 0.1 M phosphate buffer [PB], pH 7.4) immediately. After several washes with 0.1 M PB, tissues were treated successively with reduced osmium tetroxide (0.8% hexacyanoferrate II and 1% OsO_4 in PB) for 2 h, and aqueous uranyl acetate (1%) in the dark. Afterward, samples were rinsed in ultrapure water, dehydrated in an ascending ethanol series (30-50-70-85-95-100%), each step for 8 min, and exchanged with pure acetone twice. After progressive infiltration with epoxy resin (EMbed 812, 14120, Electron Microscopy Science, USA), samples were embedded and polymerized at 65°C for 24 h. Resin blocks were trimmed and cut using an ultramicrotome (UC7, Leica Microsystems, Germany) equipped with a diamond knife (Diatome, Switzerland). Serial sections (75 nm) were collected on copper, single-slot, formvar-coated grids, and poststained with 2% uranyl acetate and lead citrate. All grids were inspected in 120 kV FEI Tecnai G2 Spirit BioTWIN transmission electron microscope (FEI Company, now Thermo Fisher Scientific, USA) fitted with Gatan 832 digital camera (Gatan, Pleasanton, CA). Images were analyzed using the Image J software to determine the number, size and area of LDs. The number and total cross-sectional area of LDs were quantified per 100 μm^2 of tissue area. The mean LD area was subsequently calculated by dividing the total LD area by the number of LDs within the same reference area. The data were analyzed blindly.

Lipidomics analysis

Left ventricular ischemic myocardial tissue was used for lipidomics analysis. After 24-h reperfusion, the heart tissue was put in liquid nitrogen until metabolites extraction. After adding 400 μL water, tissues were homogenized (45 Hz, 4 min) and sonicated (5 min) in ice-water bath. The 150 μL homogenate was mixed with 50 μL water, and then 480 μL extract solution (MTBE: methanol = 5: 1) containing internal standard was added. After 30 s vortex, the samples were sonicated (10 min, ice bath), incubated (-40°C, 1 h), and centrifuged at 3000 rpm for 15 min at 4 °C. This step was repeated once. Then the supernatants were combined and dried in a vacuum concentrator at 37 °C. the samples were vortexed for 30 s and sonicated for 10 min in ice-water

bath, and 30 μL of supernatant was transferred to a fresh glass vial for lipid measurements by high-performance liquid chromatography (Biotree, Shanghai, China).[6] The quality control (QC) sample was prepared by mixing an equal aliquot 10 μL of the supernatants from all of the samples.

RNA-seq (second-generation sequencing)

RNA sequencing was performed as previously described.[7] Total RNA from the left ventricular ischemic myocardial of WT or Seipin KO mice subjected to I/R was extracted using the RNeasy Mini Kit (QIAGEN) according to the manufacturer's specification.

The sequencing data were filtered with SOAPnuke (v1.4.0), and the clean reads were mapped to the reference genome (mm10) using HISAT2 (v2.1.0). Bowtie2 (v2.2.5) was applied to align the clean reads to the gene set, a database for this organism built by BGI (Beijing Genomic Institute,China), then expression level of gene was calculated by RSEM (v1.2.8). Essentially, differential expression analysis was performed using the DESeq2 (v1.4.5) with Q value $\leq$ 0.05. Clean reads were aligned to the PacBio reference sequence, taking the comparison ratio and the distribution of the reference sequence as conditions for further.

RNA interference-mediated gene silencing

For gene-silencing assays, siRNAs were designed using the Invitrogen website. Cardiomyocytes were transfected with siRNAs using Lipofectamine RNAiMAX (13778075, Invitrogen, USA) following the manufacturer's instructions.[8] The efficiency of gene knockdown was assessed 48 h after siRNA transfection by qPCR. The siRNA sequences were listed in **Supplemental Table 1**.

Quantitative real-time PCR

Total RNA was extracted from tissues and NRVMs using Trizol reagent (Invitrogen, USA), the concentration of RNA was measured using the Nanodrop 2000 (Thermo Fisher Scientific, USA) and first-strand cDNA was generated using the TransScript First-Strand cDNA Synthesis SuperMix kit (TransGen Biotech, China).[9] Quantitative real-time PCR (qPCR) was performed

using the following primers, Luminaris qPCR SuperMix (Life Technologies) and a Roche Light Cycler 480. Each experiment was performed in duplicate and normalized to housekeeping gene as previously described. Detailed qPCR primers were in **Supplemental Table 2**. The results are represented by the ratio of the value to the control group value.

Chromatin immunoprecipitation (ChIP)- qPCR assay

ChIP was performed to assess the binding of USF1 to the Seipin promoter in mouse hearts. In brief, hearts from 8-week-old mice were cross-linked with 1% formaldehyde for 10 minutes at room temperature and the reaction was quenched with glycine. The tissues were then homogenized, and nuclei were isolated. Chromatin was sheared by sonication to an average fragment size of 200–500 bp. For each immunoprecipitation reaction, 20 μg of sheared chromatin was incubated overnight at 4°C with 3 μg of anti-USF1 antibody (ab125020, Abcam) or control normal IgG (30000-0-AP, Proteintech). Antibody-chromatin complexes were captured using Protein A/G Magnetic Beads, followed by extensive washing. The cross-links were reversed, and the immunoprecipitated DNA was purified. Enrichment of specific Seipin promoter regions was quantified by qPCR using the primers listed below. The results are presented as fold enrichment relative to the IgG control.

The sequences of specific primers to Seipin promoter binding region were: site1, forward: TTGTGTCTGGTGCATGCAGA, reverse: CCAGGCCTAGCAACTCGTAC; site2, forward: AAAACTAGAACAGCCGGGCA, reverse: ACTTTGTAGACCAGGCTGGC; site3, forward: GCCGGGATCTCACTCTAGGA, reverse: CTTGGAGCACCCTGTTCCTT.

Western blots

Total protein was extracted from mouse myocardial tissues or NRVMs using RIPA solution, and the protein content was determined by a BCA protein assay (Thermo Fisher Scientific, USA), as previously described.[9] The denatured proteins were separated by sodium dodecyl sulfate-polyacrylamide gel electrophoresis (SDS-PAGE). ECL chemiluminescence was used to detect the bands with an imaging system (molecular imager, ChemiDoc XRS, Bio-Rad, USA), and the densities of the bands were also determined semiquantitatively using the same system.

The examined protein levels were normalized to that of GAPDH. The results are represented by the ratios of the values of the experimental groups to those of the control group. Antibodies used in this study are summarized as follows: Flag (F1804, Sigma, USA), GRP78 (ab21685, Abcam, USA), Bax (ab32503, Abcam, USA), Bcl-2(ab32124, Abcam, USA), USF1 (ab125020, Abcam, USA), GAPDH (ABS16, Millipore, USA), β-tubulin (BE0025, Bioeasy, China), and appropriate horseradish peroxidase-conjugated secondary antibody (ZSGB-BIO, China).

Supplemental Table 1. The sequences of the siRNAs used in this experiment

Gene	Direction	Sequences 5'-3’
Seipin	Forward	GGUUCUUUCUACUACUCCU
	Reverse	AGGAGUAGUAGAAAGAACC
Hsd17b7	Forward	CGUUCACUGUGACACCGUA
	Reverse	UACGGUGUCACAGUGAACG
Plin4	Forward	GGACACCAGCAAGAGUGUA
	Reverse	UACACUCUUGCUGGUGUCC
Hilpda	Forward	CAACAUCAAGUGUCUCCAA
	Reverse	UUGGAGACACUUGAUGUUG
USF1	Forward	GCUCGACAAUGACGUGCUU
	Reverse	AAGCACGUCAUUGUCGAGC
DBP	Forward	GUCGAGAGACGCAAGAAGA
	Reverse	UCUUCUUGCGUCUCUCGAC
PBX1	Forward	GAGCGGAUGGUCAGCAUUA
	Reverse	UAAUGCUGACCAUCCGCUC

Supplemental Table 2. The sequences of the qPCR primers used in this experiment

Gene	Species	Direction	Sequences 5'-3’
Seipin	mice	Forward	GGCTCCTTCTACTACTCCTACA
		Reverse	CCGATCACGTCCACTCTT
IL-1β	mice	Forward	AGGCTCCGAGATGAACAA
		Reverse	AAGGCATTAGAAACAGTCC
IL-6	mice	Forward	TTCTTGGGACTGATGCTG
		Reverse	CTGGCTTTGTCTTTCTTGTT
NOX2	mice	Forward	ACTCCTTGGGTCAGCACTGG
		Reverse	GTTCCTGTCCAGTTGTCTTCG
p22 phox	mice	Forward	TGCCAGTGTGATCTATCTGCT
		Reverse	TCGGCTTCTTTCGGACCTCT
GAPDH	mice	Forward	TGGATTTGGACGCATTGGTC
		Reverse	TTTGCACTGGTACGTGTTGAT
Seipin	rat	Forward	TTGCCAATGTCTCGCTGACT
		Reverse	AGTGGAGATGATTCGGCCAC
IL-1β	rat	Forward	TGACCTGTTCTTTGAGGCTGAC
		Reverse	CATCATCCCACGAGTCACAGAG
IL-6	rat	Forward	AGGATACCACCCACAACAGACC
		Reverse	TTGCCATTGCACAACTCTTTTC
NOX2	rat	Forward	CTTTAGCATCCATATCCGCATT
		Reverse	GACTGGTGGCATTGTCACAATA
p22 phox	rat	Forward	CCTCCACTTACTGCTGTCCG
		Reverse	TGGTAGGTGGCTGCTTGATG
Hsd17b7	rat	Forward	ATGCGTTCACTGTGACACCGTAC
		Reverse	CTAATCCTGTGGTGCCGCTCAAG
Plin4	rat	Forward	GTGGCAGATTCCTGGGGAAA
		Reverse	CTGCCTCCGAAGTCACAAGG
Hilpda	rat	Forward	TAAACCTCTACGTGCTGGGG

		Reverse	GCTGTGTGTTGGCTAGGTGA
USF1	rat	Forward	AAGTCAGAGGCTCCCAGGA
		Reverse	CGGCGCTCCACTTCGTTAT
DBP	rat	Forward	AGACTTACACCTGACACCCC
		Reverse	GATTGTGTTGATGGAGGCGG
PBX1	rat	Forward	AAGTGCGGCATCACAGTCTC
		Reverse	GAAGGGTATCCACCCGCTG
Ldha	rat	Forward	ACGCAGACAAGGAGCAGTGGAA
		Reverse	ATGCTCTCAGCCAAGTCTGCCA
HK2	rat	Forward	CTCCATCCCACAGGAGGTTA
		Reverse	GCTTCCTTCAGCAAGGTGAC
Pdk4	rat	Forward	CCGCATTTCTACTCGGATGC
		Reverse	AGGCATCTTGGACTACTGCT
Pdha1	rat	Forward	TGCAGAGCTAACAGGACGAA
		Reverse	TTGGCGTACATGTGCATTGA
Acsl1	rat	Forward	AGTACCTGATGTGGAGATCCTACCTTC
		Reverse	TCTTCCCAAGTTTCACCATGTCTTCC
Cpt1a	rat	Forward	CAGGAGAGTGCCAGGAGGTCATAG
		Reverse	TGCCGAAAGAGTCAAATGGGAAGG
CerS6	rat	Forward	CAGACCTGAAGAACACGGAGGA
		Reverse	GTCCATTGGCTTGGATGTTGAGG
Degs1	rat	Forward	GCCTCTGAACTTGCTCACCTTC
		Reverse	TGATCCAGGAGTTGTAGTGCGG
Agpat2	rat	Forward	AGCGGACAGAAGAAACTGGAGG
		Reverse	TTAGCTCACGCTTGGCGATCTG
Lpin1	rat	Forward	CCTGGAAATGCTCTGGCTATGG
		Reverse	CGCTGTGAATGGTCTGGAAGT
GAPDH	rat	Forward	TGATGACATCAAGAAGGTGGTGAAG
		Reverse	TCCTTGGAGGCCATGTAGGCCAT

Reference

[1] P. Lai, G. Miao, Y. Zhao, et al., SR-A3 suppresses AKT activation to protect against MAFLD by inhibiting XIAP-mediated PTEN degradation, *Nat Commun* **2025**, *16* (1), 2430.

[2] X. Wu, X. Liu, H. Wang, et al., Seipin Deficiency Accelerates Heart Failure Due to Calcium Handling Abnormalities and Endoplasmic Reticulum Stress in Mice, *Front Cardiovasc Med* **2021**, *8*, 644128.

[3] J. Zhang, R. Liang, K. Wang, et al., Novel CaMKII-δ Inhibitor Hesperadin Exerts Dual Functions to Ameliorate Cardiac Ischemia/Reperfusion Injury and Inhibit Tumor Growth, *Circulation* **2022**, *145* (15), 1154-1168.

[4] T. Zhang, Y. Zhang, M. Cui, et al., CaMKII is a RIP3 substrate mediating ischemia- and oxidative stress-induced myocardial necroptosis, *Nat Med* **2016**, *22* (2), 175-182.

[5] H. Zhang, T. Sun, X. Jiang, et al., PEDF and PEDF-derived peptide 44mer stimulate cardiac triglyceride degradation via ATGL, *J Transl Med* **2015**, *13*, 68.

[6] W. Xiang, R. Shi, X. Kang, et al., Monoacylglycerol lipase regulates cannabinoid receptor 2-dependent macrophage activation and cancer progression, *Nat Commun* **2018**, *9* (1), 2574.

[7] Y. Yao, F. Li, M. Zhang, et al., Targeting CaMKII-δ9 Ameliorates Cardiac Ischemia/ Reperfusion Injury by Inhibiting Myocardial Inflammation, *Circ Res* **2022**, *130* (6), 887-903.

[8] M. Zhang, H. Gao, D. Liu, et al., CaMKII-δ9 promotes cardiomyopathy through disrupting UBE2T-dependent DNA repair, *Nat Cell Biol* **2019**, *21* (9), 1152-1163.

[9] H. Wang, X. Huang, P. Xu, et al., Apolipoprotein C3 aggravates diabetic nephropathy in type 1 diabetes by activating the renal TLR2/NF-κB pathway, *Metabolism* **2021**, *119*, 154740.
