# Supplementary material for: Seipin‐Mediated Lipid Droplet Formation in Cardiomyocytes Ameliorates Cardiac Ischemia/Reperfusion Injury
Source: Adv Sci (Weinh). 2025 Nov 19;13(6):e10203. doi: 10.1002/advs.202510203 (PMC12866716; doi:10.1002/advs.202510203)

Figure 4A

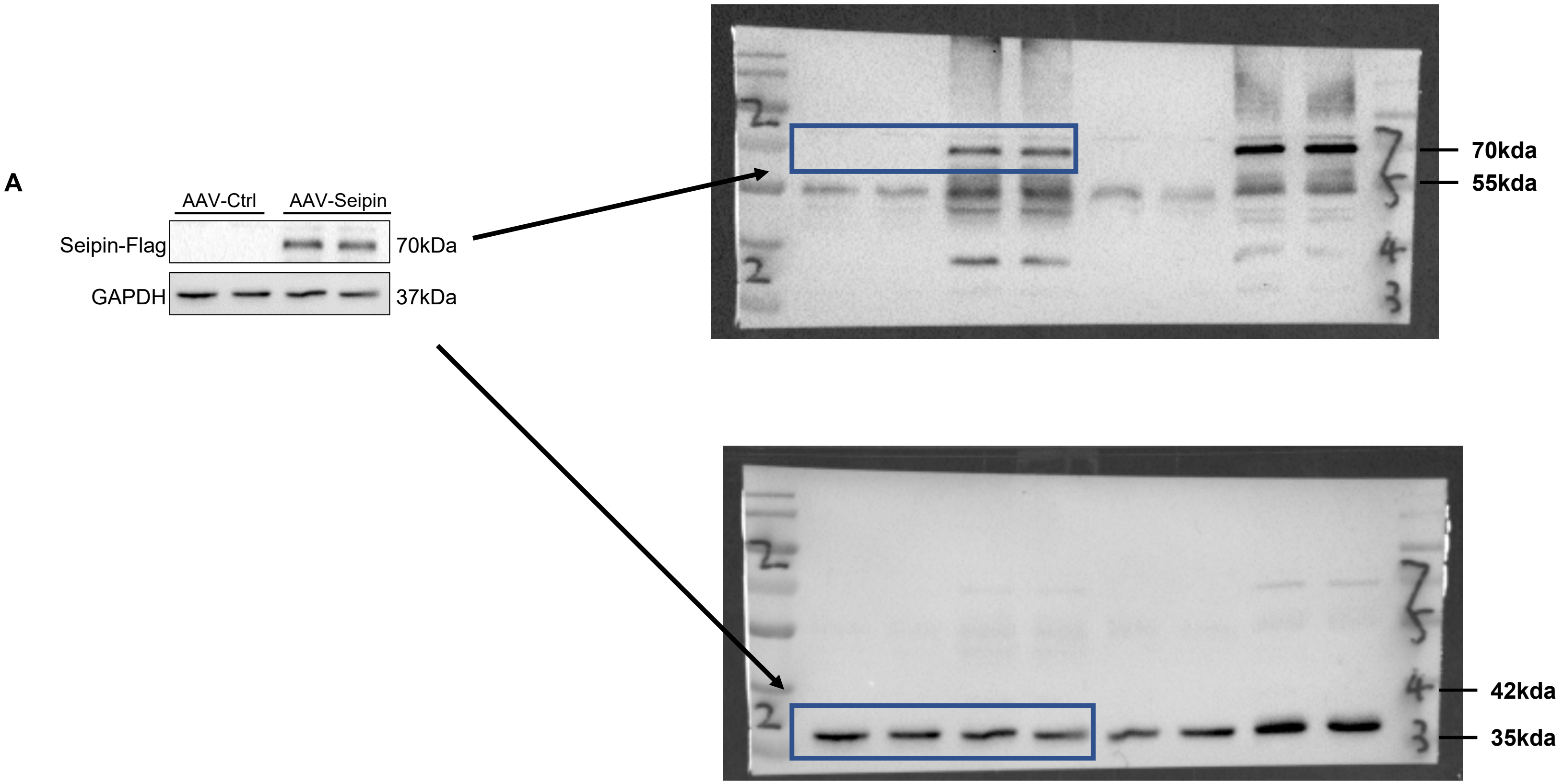
A
AAV-Ctrl
AAV-Seipin
Seipin-Flag
70kDa
GAPDH
37kDa
70kda
55kda
42kda
35kda

Figure 6A

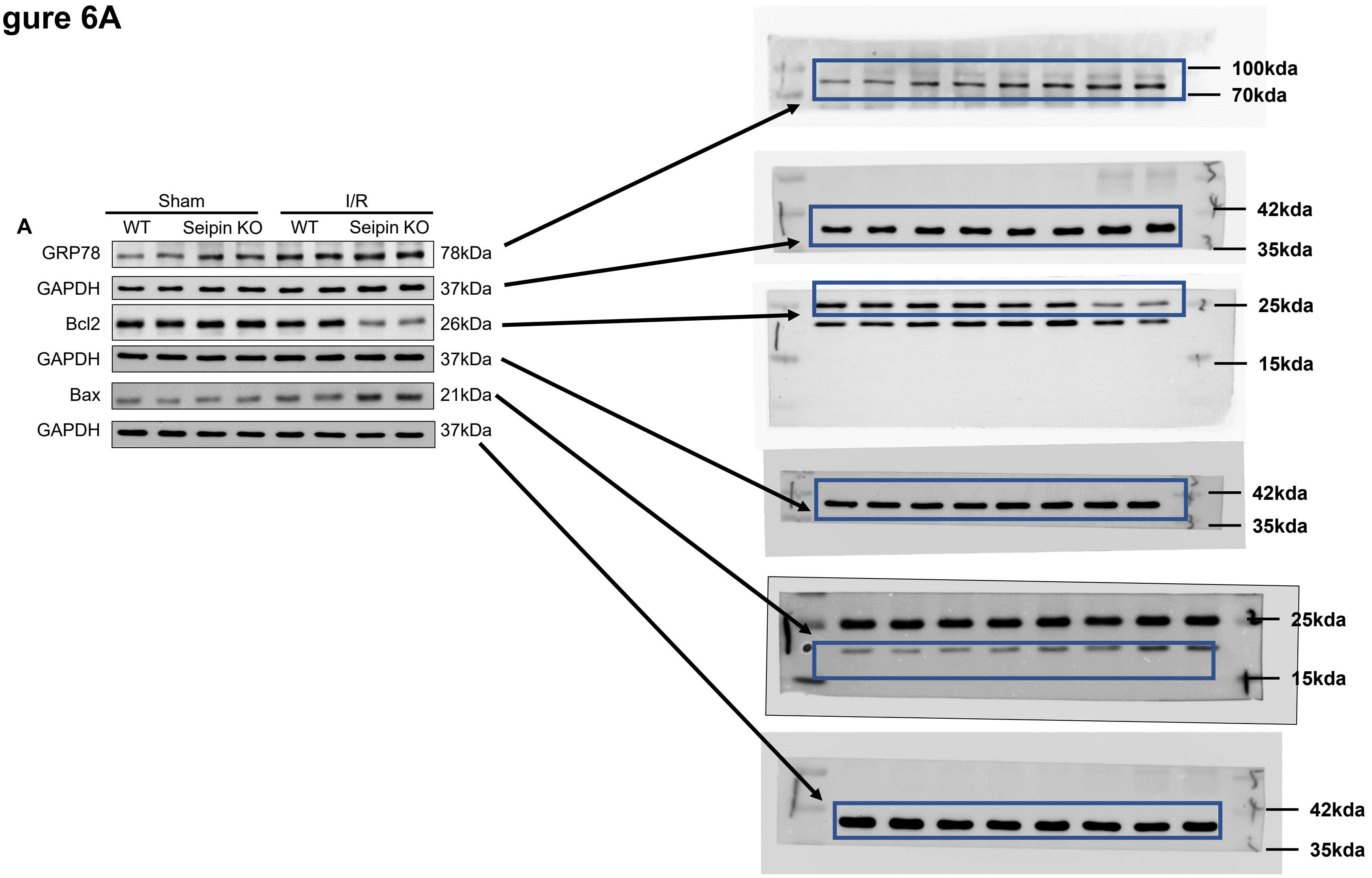
A
Sham
I/R
WT
Seipin KO
WT
Seipin KO
GRP78
GAPDH
Bcl2
GAPDH
Bax
GAPDH
78kDa
37kDa
26kDa
37kDa
21kDa
37kDa
100kda
70kda
42kda
35kda
25kda
15kda
42kda
35kda
25kda
15kda
42kda
35kda

Other blots used for quantification for Figure 6A.
The sample order is Sham-WT, Sham-Seipin KO, I/R-WT and I/R-Seipin KO with n=2 in each group.

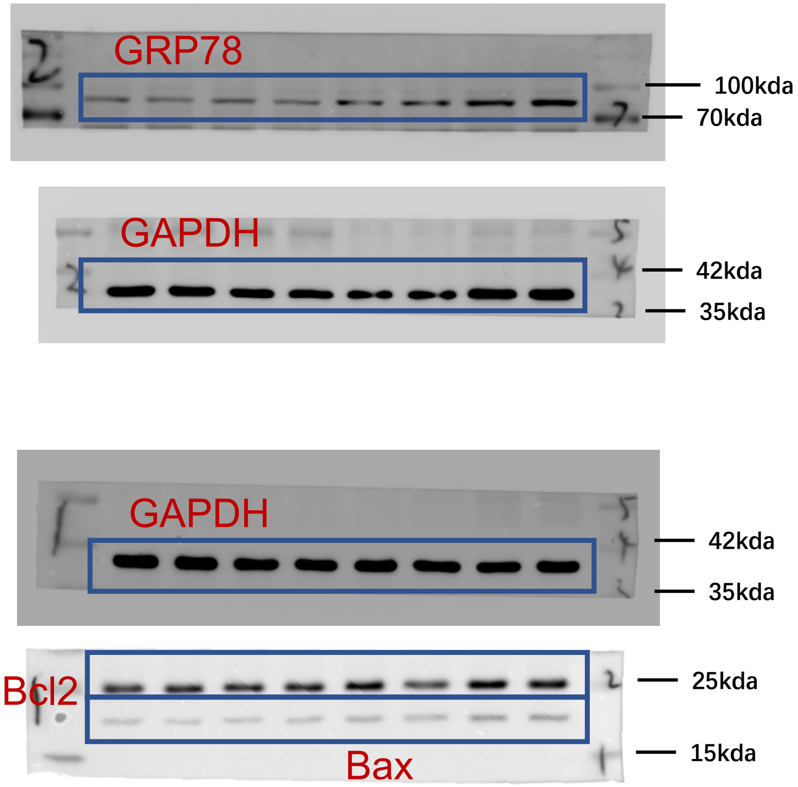

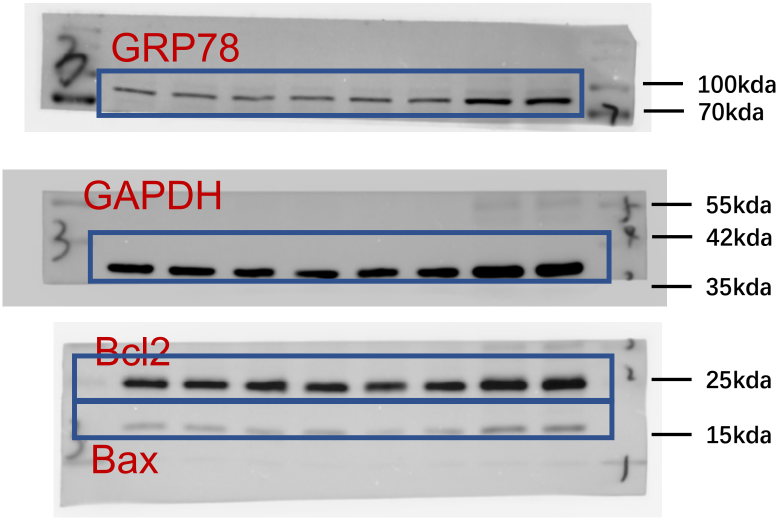

Figure 6G

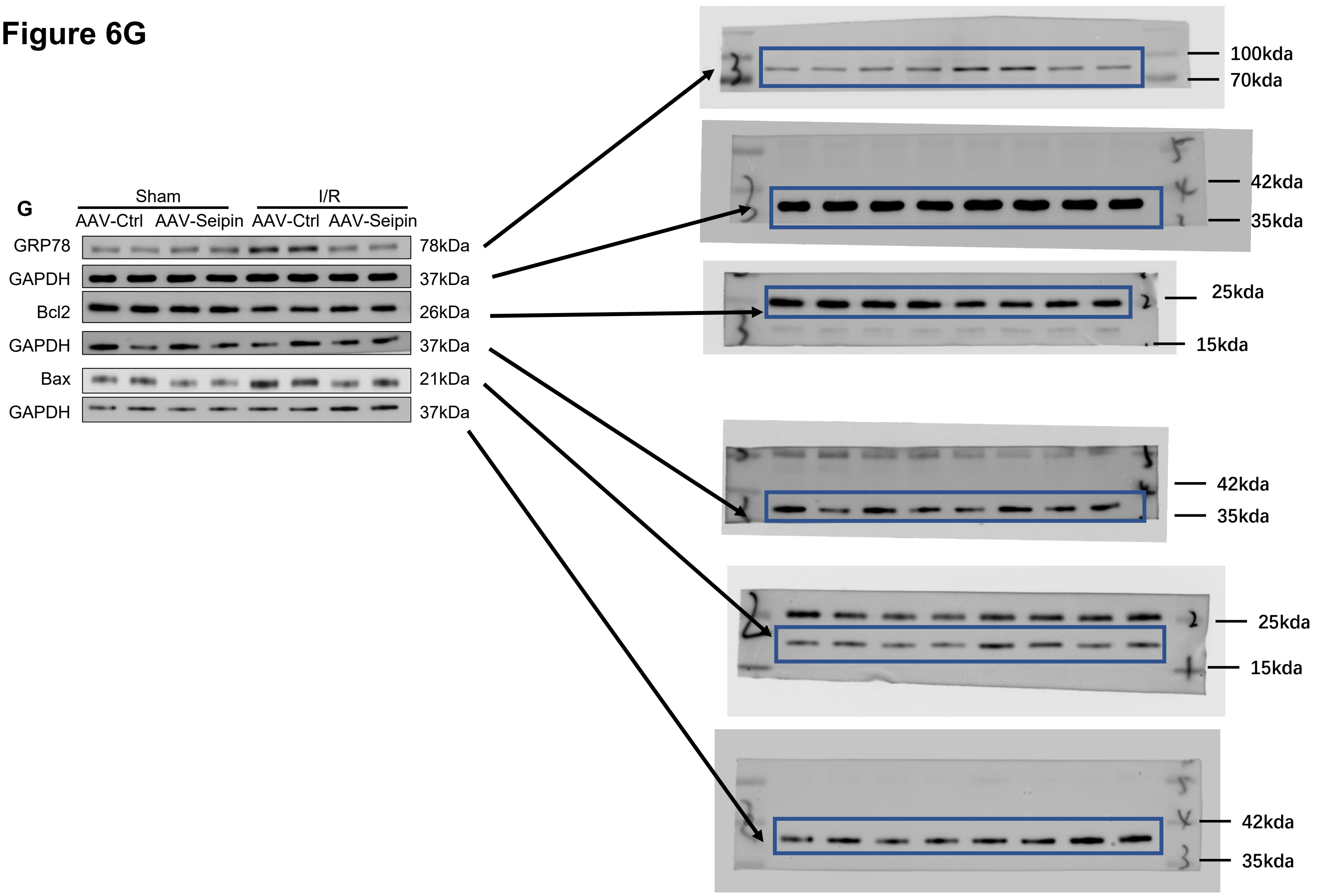

G
Sham
I/R
AAV-Ctrl
AAV-Seipin
AAV-Ctrl
AAV-Seipin
GRP78
GAPDH
Bcl2
GAPDH
Bax
GAPDH
78kDa
37kDa
26kDa
37kDa
21kDa
37kDa
100kda
70kda
42kda
35kda
25kda
15kda
42kda
35kda
25kda
15kda
42kda
35kda

Other blots used for quantification for Figure 6G.
The sample order is Sham-AAV-Ctrl, Sham-AAV-Seipin, I/R-AAV-Ctrl and I/R-AAV-Seipin with n=2 in each group.

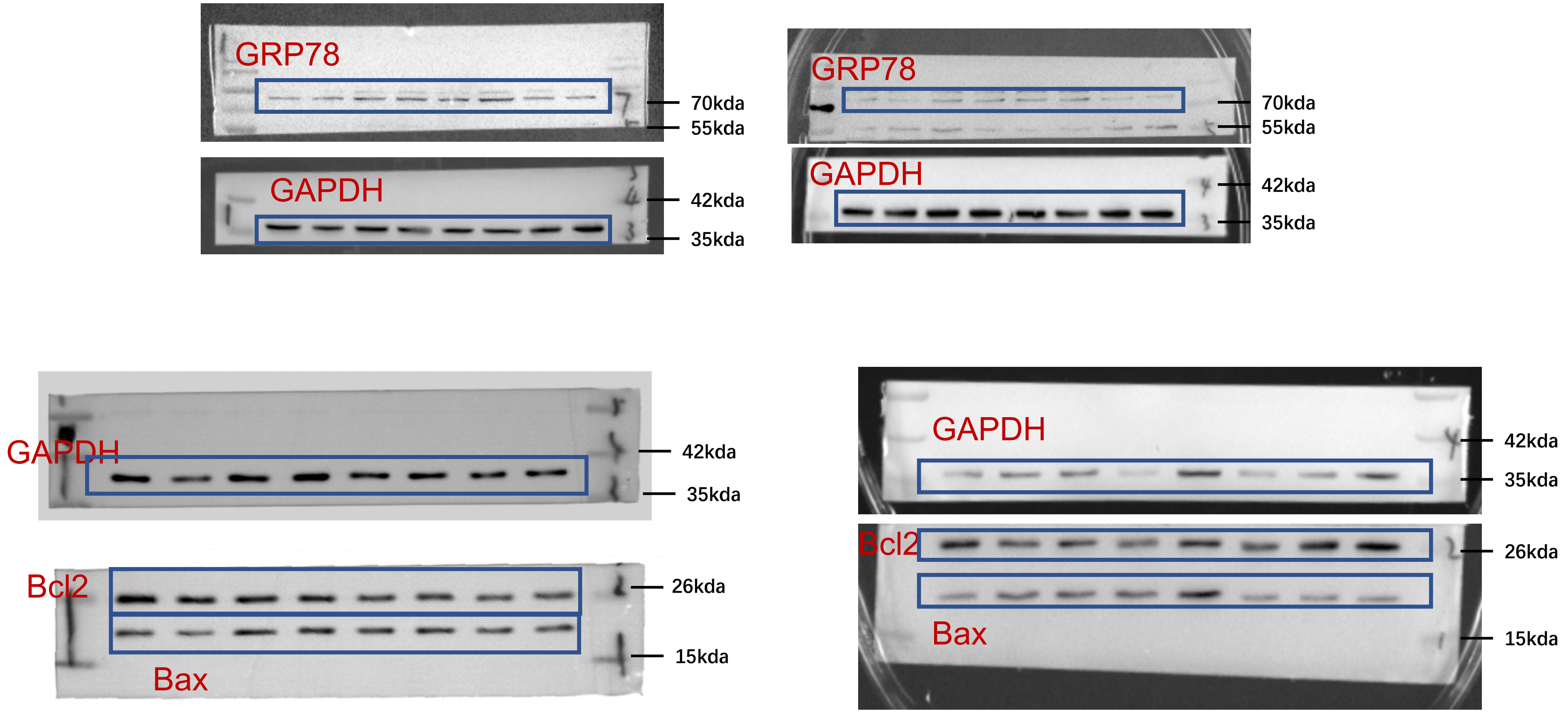

Figure 7I

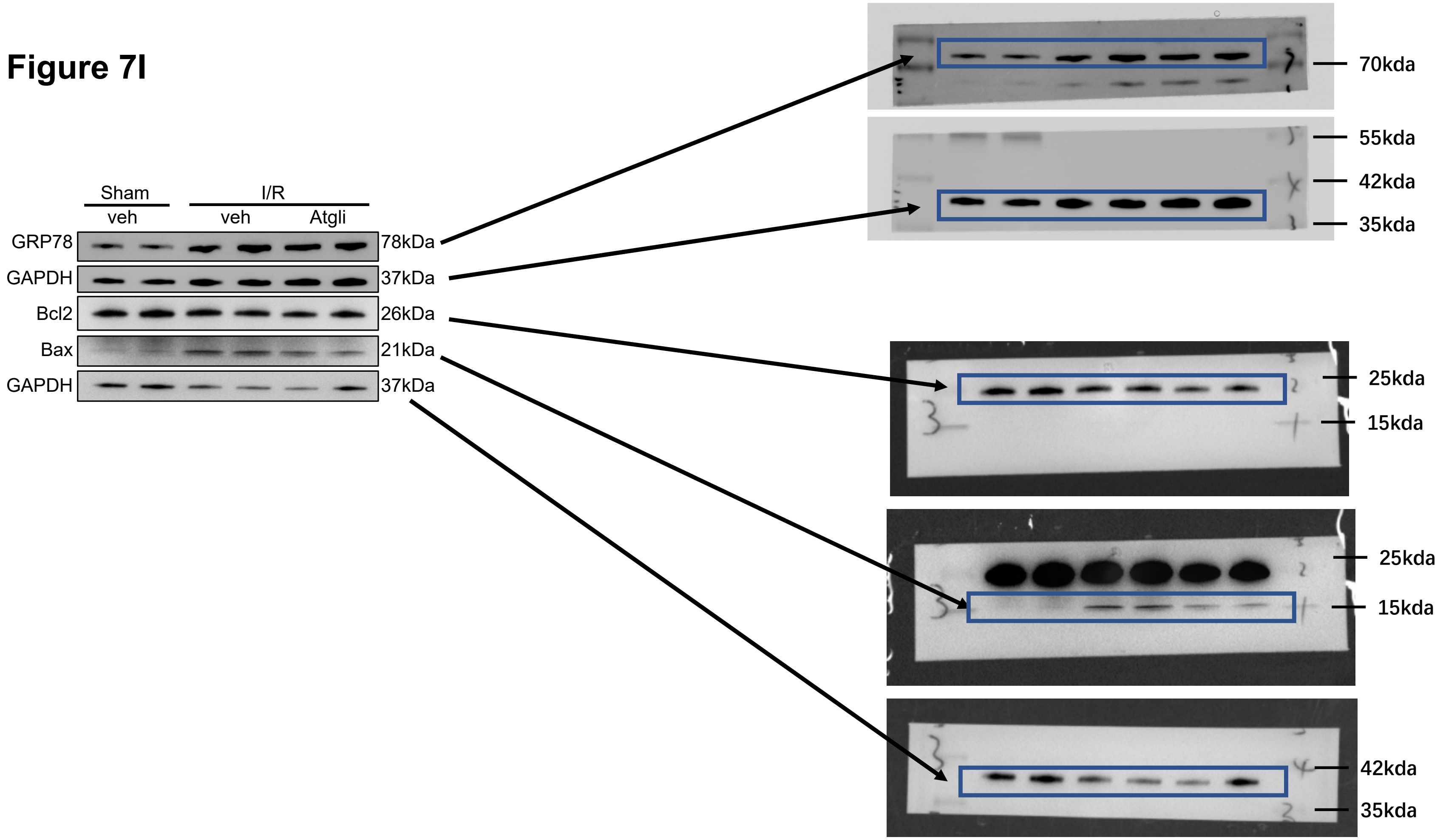

Sham
I/R
veh
veh
Atgli
GRP78
GAPDH
Bcl2
Bax
GAPDH
78kDa
37kDa
26kDa
21kDa
37kDa
70kda
55kda
42kda
35kda
25kda
15kda
25kda
15kda
42kda
35kda

Other blots used for quantification for Figure 7I.
The sample order is Sham-vehicle, I/R- vehicle and I/R-AAV-Atglistatin with n=2 in each group.

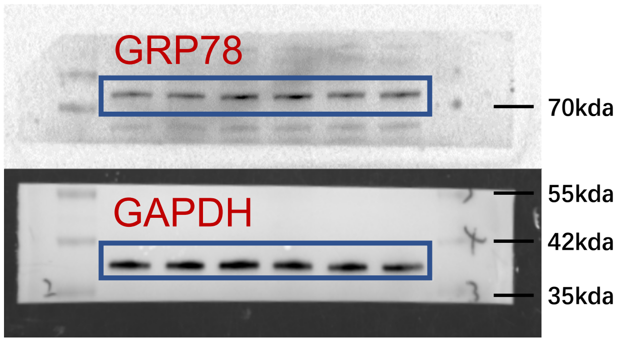

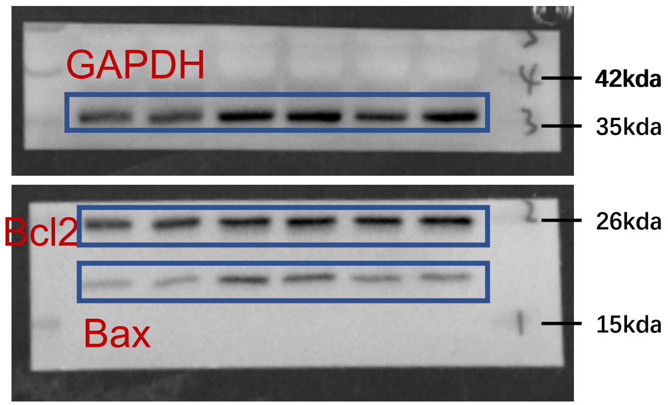

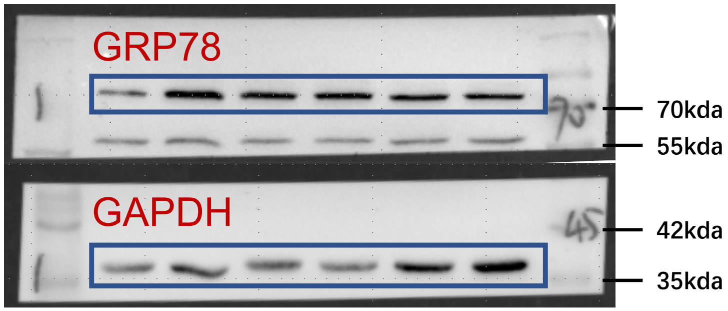

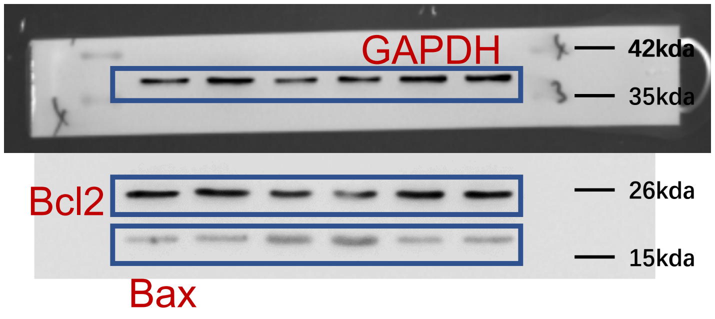

Figure 8F

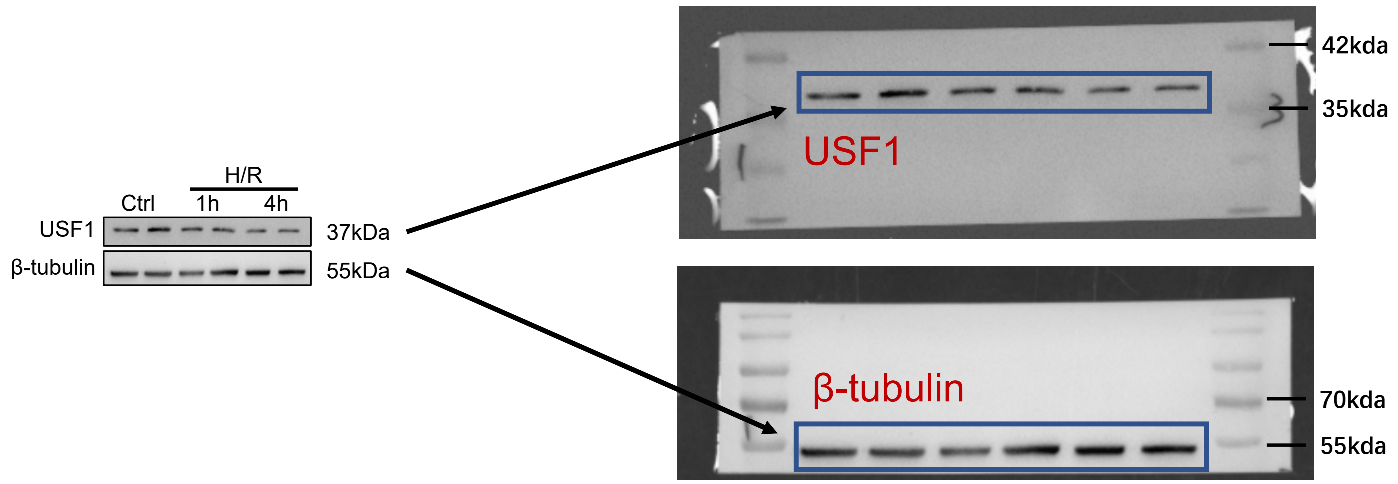

Other blots used for quantification for Figure 8F.
The sample order is Ctrl, H/R-1h and H/R-4h with n=3 in each group.

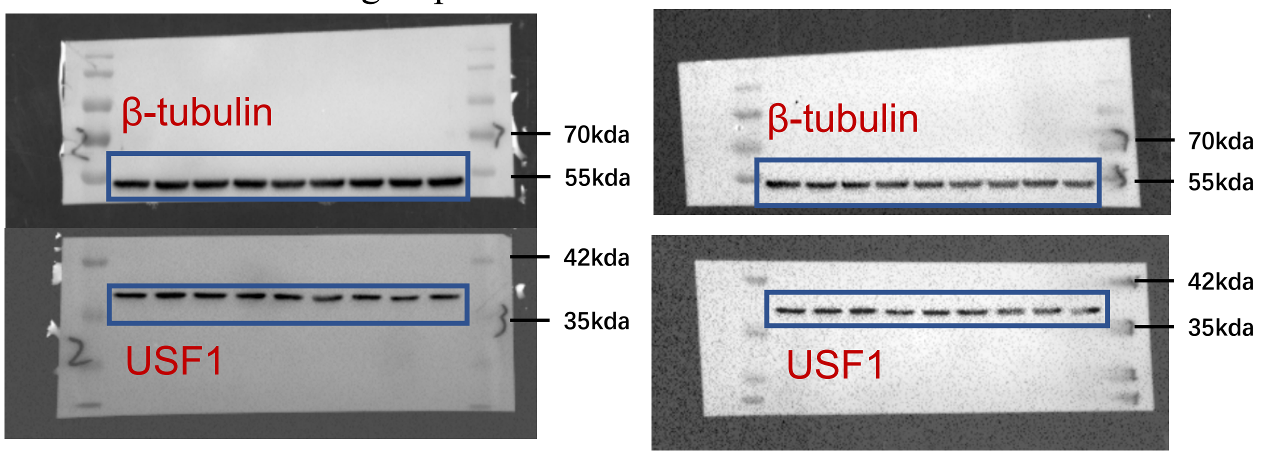

Supplement Figure S3

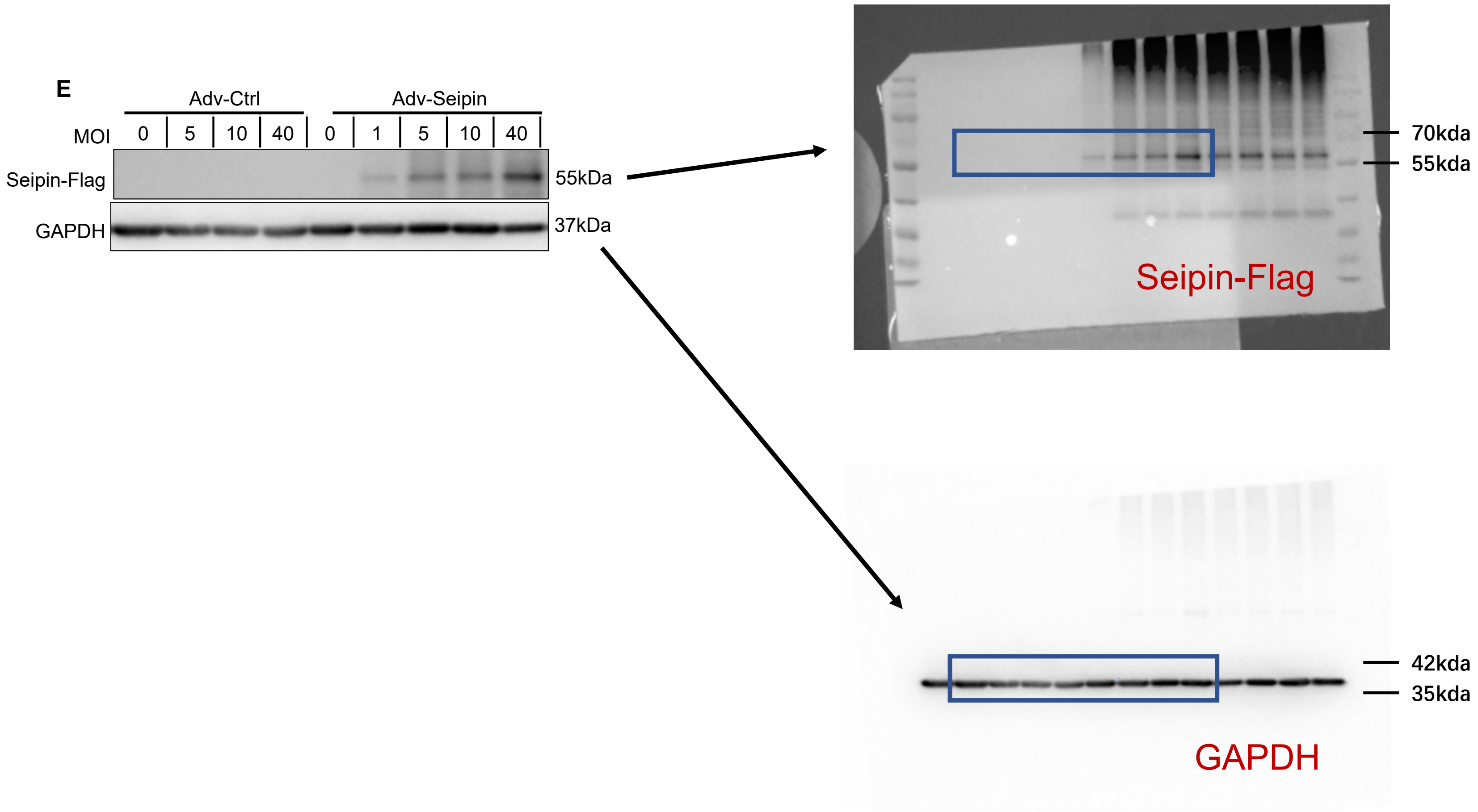

Supplement: Supplementary file 2 — Supporting Information [file ADVS-13-e10203-s001.pdf]
